# Recurrent synapses between CO_2_-sensitive olfactory sensory neurons enable robust CO_2_ detection in *Aedes aegypti* mosquitoes

**DOI:** 10.1371/journal.pbio.3003959

**Published:** 2026-09-10

**Authors:** Jialu Bao, Wesley Alford, Avinash Khandelwal, Laurel Walsh, George Lantz, Santiago Poncio, Laia Serratosa Capdevila, Yervand Azatian, Brian DePasquale, David G. C. Hildebrand, Meg A. Younger, Wei-Chung Allen Lee

**Affiliations:** 1 Department of Neurobiology, Harvard Medical School, Boston, Massachusetts, United States of America; 2 Molecules, Cells, and Organisms Training Program, Department of Molecular and Cellular Biology, Harvard University, Cambridge, Massachusetts, United States of America; 3 Department of Biology, Boston University, Boston, Massachusetts, United States of America; 4 Department of Biomedical Engineering, Boston University, Boston, Massachusetts, United States of America; 5 Laboratory of Neural Systems, The Rockefeller University, New York, New York, United States of America; 6 Department of Vision Sciences, College of Optometry, University of Houston, Houston, Texas, United States of America; 7 F.M. Kirby Neurobiology Center, Boston Children’s Hospital, Harvard Medical School, Boston, Massachusetts, United States of America; University of Michigan, UNITED STATES OF AMERICA

## Abstract

The mosquito *Aedes aegypti*’s human host-seeking behavior depends on the integration of multiple sensory cues. One of these cues, carbon dioxide (CO_2_), gates odorant and heat pathways and activates host-seeking behavior. The neuronal circuits underlying processing of CO_2_ information remain unclear. We used automated serial-section transmission electron microscopy (EM) to image and reconstruct the circuitry of the glomeruli that are innervated by the *Ae. aegypti* maxillary palp, including the glomerulus that responds to CO_2_. Notably, CO_2_-sensitive olfactory sensory neurons (OSNs) make high levels of recurrent synaptic connections with one another, while making a low density of feedforward synapses. At some of these contacts between CO_2_ OSNs, we observe ribbon-like presynaptic structures, which may further enhance recurrent signaling. We compared both feedforward and recurrent connectivity with all olfactory glomeruli in *Drosophila melanogaster,* and we found more recurrent connections between the *Ae. aegypti* CO_2_-responsive OSNs than in any *D. melanogaster* glomeruli. We developed a computational circuit model that demonstrates recurrent synapses are necessary for robust CO_2_ detection under normal physiological conditions. Together, elevated levels of recurrent connectivity and ribbon-like structures may amplify sensory information detected by CO_2_-sensitive OSNs to support mosquito activation and sensitization by CO_2_, even in the presence of high levels of other odorants in the environment. We propose that this circuit organization supports the salience of CO_2_ as a mosquito host cue.

## Abstract 2

Carbon dioxide is among the most important cues used by Aedes aegypti mosquitoes when searching for a human blood meal. This study shows that the wiring of the neurons that sense carbon dioxide differs from the wiring in Drosophila to support robust detection of this ethologically key host cue.

## Introduction

**Fig 1:**
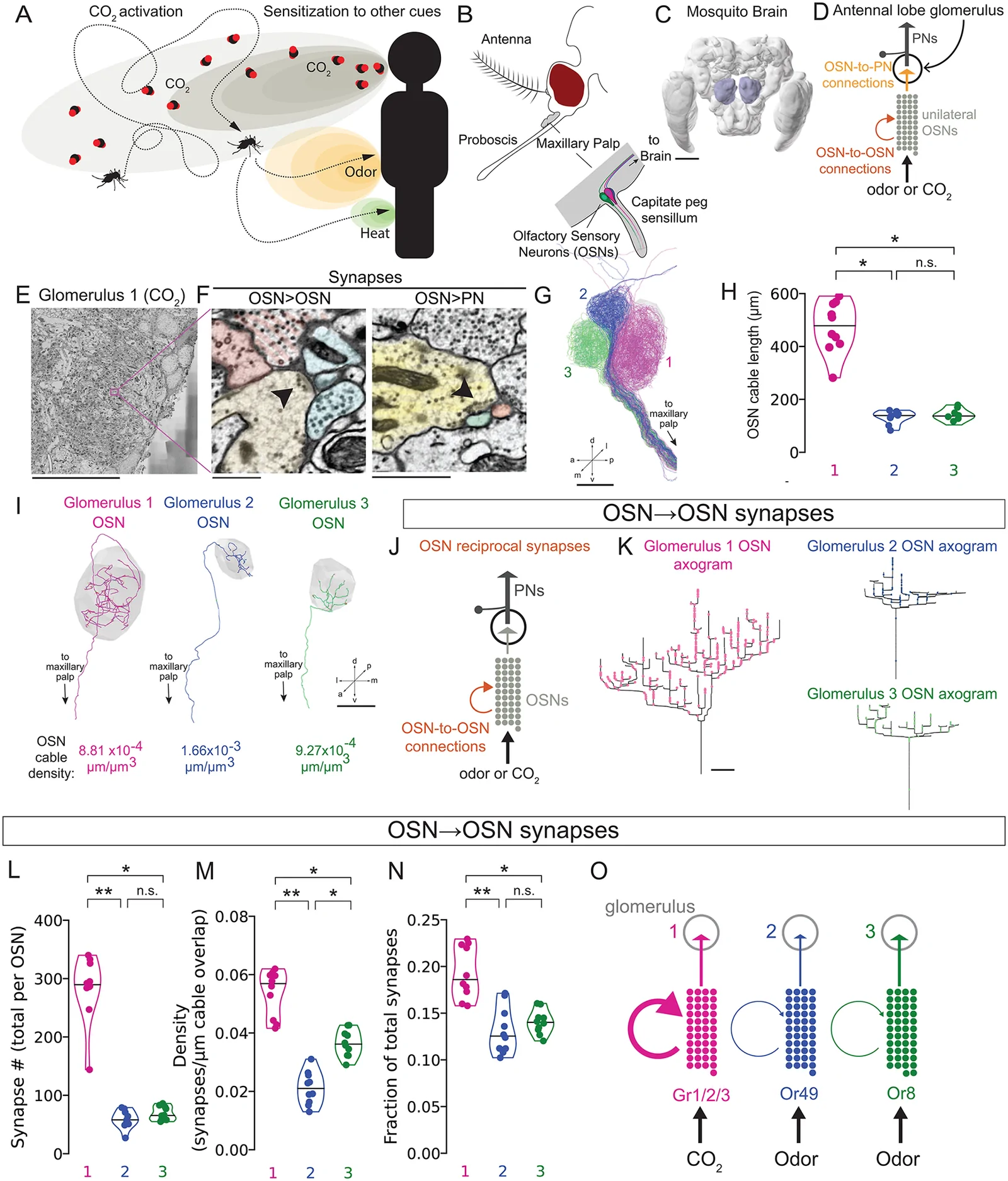
EM reconstruction of *Aedes aegypti* mosquito maxillary palp glomeruli. **(A)** Schematic of CO_2_ activation and sensitization in *Ae. aegypti* host-seeking. (left) Quiescent adult female mosquitoes become activated upon CO_2_ detection. (right) Sensitization to additional host cues (e.g., odor, heat, etc.) drives host-seeking behavior. **(B)** Diagrams (top) of an adult female *Ae. aegypti* head and (bottom) a capitate peg sensillum on maxillary palp (gray) which contain dendritic processes of olfactory sensory neurons (OSNs), including CO_2_-sensitive *Gr3*-expressing OSNs. OSN axons project centrally to the antennal lobes in the brain. **(C)** Volumetric rendering of the mosquito brain neuropils including the antennal lobes (light blue) [16,17]. Scale bar 100 µm. **(D)** Schematic of an antennal lobe glomerulus and the circuit components analyzed here. Multiple OSNs selective to a chemosensory cue (e.g., odor or CO_2_) converge on a glomerulus where they make synapses onto a cognate projection neuron (PN). OSNs can also make reciprocal synapses onto other OSNs. **(E)** Coronal 40 nm-thick section through maxillary palp Glomerulus 1, acquired and aligned using large-scale, tape-based transmission electron microscopy (EM) [18]. Scale bar 25 µm. Magenta inset expanded in **(F)** (left) Example OSN (yellow) to OSN (blue) synapse (black arrowhead). (right) Polyadic synapse (black arrowhead) in a Glomerulus 1 from an OSN (yellow) to a postsynaptic multiglomerular neuron (MG; red) and uniglomerular projection neuron (uPN; green). Scale bar 1 µm. **(G)** Sagittal view of reconstructed maxillary palp nerve OSNs: Glomerulus 1 (pink), 2 (blue), and 3 (green). Scale bar 25 µm. a, anterior; d, dorsal; l, lateral; m, medial; p, posterior; v, ventral. **(H)** Cable length of OSNs (Kruskal–Wallis test (two-tailed), *p* = 6.20 × 10^−5^, pairwise comparisons using Dunn’s post-hoc test with Bonferroni correction: n.s., *p* > 0.05; * *p* ≤ 0.01, ** p ≤ 0.001). **(I)** Coronal view of individual OSNs that innervate Glomerulus 1, 2, or 3 (gray surface meshes). Scale bar 25 µm. **(J)** Schematic with reciprocal OSN-to-OSN connections highlighted. **(K)** Axograms of representative OSNs from each glomerulus and their reciprocal output synapses to other OSNs. Scale bar 25 µm. **(L)** Total number of outgoing OSN-to-OSN synapses contained within Glomerulus 1, 2, and 3 for 10 fully reconstructed OSNs (Kruskal–Wallis test (two-tailed), *p* = 1.28 × 10^−5^, pairwise comparisons using Dunn’s post-hoc test with Bonferroni correction: n.s., *p* > 0.05, * *p* ≤ 0.05; ** *p* ≤ 0.001). **(M)** Synapse density of outgoing OSN-to-OSN synapses per micrometer of cable overlap (length of postsynaptic OSN axon within 2 µm of presynaptic OSN axon, see Methods) (Kruskal–Wallis test (two-tailed), *p* = 0.0003, pairwise comparisons using Dunn’s post-hoc test with Bonferroni correction: n.s., *p* > 0.05; * *p* ≤ 0.05; ** *p* ≤ 0.001). **(N)** Reciprocal OSN-to-OSN synapses as a fraction of overall OSN output synapses (Kruskal–Wallis test (two-tailed), *p* = 0.0002, pairwise comparisons using Dunn’s post-hoc test with Bonferroni correction: n.s., *p* > 0.05, * *p* ≤ 0.01, ** *p* ≤ 0.001). (G) Schematic of reciprocal connectivity with arrow thickness reflecting synapse number. The data underlying this Figure can be found in S1–S4 Data.

Mosquito-borne disease claims over half a million lives every year. The mosquito *Aedes aegypti* is a globally distributed disease vector responsible for thousands of deaths annually [1], with fatalities projected to increase over time [2,3]. Adult female mosquitoes innately seek blood to obtain the protein required for egg maturation [4] and they integrate a range of sensory cues to detect their preferred host, humans. These cues include skin-derived odorants [5–7], body heat [8,9], water vapor [10,11], visual cues [8,12], and carbon dioxide (CO_2_) [6,7,13]. The host cue CO_2_ drives notable changes in behavior, including increased locomotion, evident from both increases in take-off and flight time, as well as increased upwind flight [6,14]. These behaviors are presumed to initiate the search for a host [6], a state change termed *activation* [6,14]. An activated mosquito investigates host cues such as body odor, heat, and visual contrast. These synergize to drive host-seeking and biting behaviors, which include landing and probing with the proboscis [6]. We refer to the priming of other sensory channels during behavioral activation as CO_2_ *sensitization* [6,15] (Fig 1A). We sought to discover how CO_2_ activation and sensitization mechanisms are reflected in the neuronal circuitry that detects CO_2_ and integrates it with other host cues.

Many principles of chemosensory wiring and coding were established in the model fly, *Drosophila melanogaster*. Olfactory sensory neurons (OSNs) in the antennae and maxillary palps [19,20] innervate ~50 glomeruli in the antennal lobes (ALs) [21,22], the first olfactory processing center of the brain. Multiple OSNs express a single olfactory receptor and all OSNs expressing the same receptor converge on a single glomerulus [19,20], which can be defined as an anatomically distinct local circuit. Local neurons (LNs) project among antennal lobe glomeruli, transforming information through a combination of excitatory and inhibitory interactions [23]. From antennal lobe glomeruli, projection neurons (PNs) send their axons to higher brain centers. Synapse-resolution reconstructions of the *D. melanogaster* olfactory system [24–26] and connectomes of the whole brain have recently been generated [27–30]. This work revealed circuit mechanisms of sensory coding [31–33], innate behavior [34,35], and learning and memory [34,36]. *Ae. aegypti* and *D. melanogaster* are both dipteran insects, but they have evolved dramatically different feeding strategies during the more than ~150-million-year timespan since they shared a common ancestor [37,38]. This has led to different specializations in chemosensory information processing. Recent work identified the co-expression of multiple olfactory receptors in many *Ae. aegypti* OSNs [39,40], and it is likely that additional differences in the chemosensory circuit architecture support their diet of primarily human blood.

Information processing in the olfactory system, like other neuronal circuits, relies on synapses, which are the main sites of information transmission between neurons. At chemical synapses, neurotransmitters are packaged into synaptic vesicles and released from specialized presynaptic active zones for detection by receptors at postsynaptic sites. Vesicular release machinery traffics vesicles to the presynaptic membrane, docks vesicles at the active zone, and drive vesicular exocytosis [41,42]. The structure of presynaptic active zones is diverse across species and synapse types, and related to functional properties of different synapses [43–46]. In insects, synapses typically have a small electron-dense structure referred to as a T-bar (which appears as a T when cut in cross-section) that promotes vesicle docking and release [45,47]. In contrast, vertebrate hair cells and neurons in the retina have large presynaptic specializations called ribbon synapses, which are elongated ribbon-shaped electron-dense structures extending intracellularly from the presynaptic active zone. Ribbon synapses are thought to support sustained and graded synaptic vesicle release [44,46]. Ribbon synapses include the vertebrate-specific protein Ribeye [48–50] and have not previously been identified in invertebrates. The existence of ribbon-like synapses in invertebrates would imply a convergently evolved mechanism for sustained or graded synaptic vesicle release across species.

In *Ae. aegypti*, the maxillary palp (Fig 1B) is the sensory appendage that contains neurons that sense CO_2_. To examine the circuit architecture that underlies CO_2_ sensitivity, we generated a synapse-resolution wiring diagram of the three glomeruli that are innervated by maxillary palp OSNs. We used automated transmission electron microscopy (EM) [18] to generate an EM volume that includes all three glomeruli innervated by the OSNs that arise from the maxillary palp. For each of the three glomeruli, we reconstructed and annotated every synapse in 10 OSNs and their cognate uniglomerular PNs (uPNs) that exit these glomeruli and project in the inner antennocerebral tract (iACT). We found that CO_2_-sensitive OSNs had high levels of recurrent connections, a motif that may be used to amplify CO_2_ detection. To compare chemosensory coding strategies in animals with divergent feeding behaviors, we analyzed the recurrent and feedforward connectivity of all *D. melanogaster* olfactory glomeruli including the CO_2_ glomerulus, Glomerulus V, using FlyWire [28]. We did not find recurrent or feedforward connectivity to be similar between CO_2_-sensitive glomeruli in *Ae. aegypti* and *D. melanogaster*. Rather, there were far more recurrent connections between CO_2_-sensitive OSNs in *Ae. aegypti* than the OSNs that innervate any glomerulus in the fly. We identified a novel structure located specifically in CO_2_-sensitive OSNs reminiscent of mammalian ribbon synapses [44,46,51–53], and propose that these ribbon-like structures may supplement excitatory axo-axonic signaling. Lastly, we adapted a biophysical model from *D. melanogaster* to include recurrent connections. Using this model and previously published measurements, we found that recurrent connections supported robust CO_2_ detection, even in the presence of strong background odors that otherwise limit CO_2_ detection sensitivity. Together, this work suggests that elevated levels of recurrent connectivity and ribbon-like synapses may amplify incoming CO_2_ cues to support mosquito activation and sensitization to CO_2_.

## Results

### EM reconstruction of olfactory sensory neurons reveals high recurrent connectivity among CO_2_ neurons

*Ae. aegypti* rely on multiple sensory cues to locate humans (Fig 1A), and some of these cues, including CO_2_, are first detected in the maxillary palp (Fig 1B) by OSNs that innervate the antennal lobes (Fig 1C and 1D). To compare the circuits that detect and process CO_2_ to those dedicated to other odorants, we set out to image and reconstruct multiple antennal lobe glomeruli with electron microscopy (EM). We used automated serial-section transmission EM [18] to image the posterior two-thirds of the antennal lobes (Fig 1C–1F) of an adult female *Ae. aegypti* brain (S1A Fig) at 4 × 4 × 40 nm^3^/voxel resolution. This region of the antennal lobe includes the three glomeruli that are innervated by olfactory sensory neurons of the maxillary palp: the CO_2_-responsive glomerulus, Glomerulus 1, and two other olfactory glomeruli, Glomeruli 2 and 3, which respond to odorants [39,54].

In order to analyze the connectivity of these circuits, we manually reconstructed neurons in Glomeruli 1, 2, and 3 on the right side of the brain (Fig 1G–1J). We identified neurons as olfactory sensory neurons (OSNs), uPNs, or multiglomerular neurons (MGs) by their morphology, axon tracts, and which glomeruli they innervated. Most MGs extended beyond the imaged volume, and we therefore could not determine their cell type. We therefore focus our analysis on OSN and uPN circuitry (see Methods for details).

To examine the morphology of individual OSNs, we first annotated the processes of every OSN projecting from the right maxillary palp nerve by creating a skeleton reconstruction of each OSN that includes all its branches (Fig 1G and 1I). We identified 136 OSNs that innervate Glomeruli 1, 2, and 3. Each capitate peg sensillum on the maxillary palp contains 3 OSNs [55], and each OSN is thought to project to a single glomerulus. We found that 45 OSNs that project to Glomerulus 1, 45 to Glomerulus 2, and 46 to Glomerulus 3. Consistent with previous work [39,54], we saw that the axonal arbors of each OSN projected to a single glomerulus (Figs 1G,1I, S3, and S4).

From this population of OSNs, we chose 10 OSNs innervating each glomerulus that represented the morphologies of the total population by visual inspection (Figs 1G,1I, and S3). We then tested whether these were representative of the morphologies of the total OSN population using NBLAST [56] and found that they were not significantly different from the overall population (Welch’s *t* test, *p* = 0.21, *p* = 0.07, *p* = 0.19 for Glomerulus 1, 2, and 3, respectively). The number of excitatory synapses between two cells is indicative of the amplitude and probability of depolarization in a postsynaptic neuron after the presynaptic neuron fires [24,57]. We therefore reconstructed these 30 OSNs (10 per glomerulus) to completion, meaning that all synapses made by these neurons onto any postsynaptic partner were annotated. We quantified the length of all neurites (cable length) and branching pattern of these fully reconstructed OSNs and found that Glomerulus 1 OSN cable length was over twice as long and their arbors were more complex compared to Glomerulus 2 or 3 OSNs (Figs 1H and S5A–S5C). Due to the large volume of Glomerulus 1, its neurite density is lower than Glomerulus 2 or 3 despite the elevated cable length and branching complexity (Fig 1H and 1I).

One mechanism for signal amplification in neural networks is recurrent or reciprocal excitatory connectivity [58,59]. We next asked if *Ae. aegypti* OSNs have recurrent connectivity that could amplify the detection of host cues. We examined recurrent connections between the OSNs that innervate each glomerulus (Fig 1J–1N). We define a recurrent synapse as any OSN-to-OSN synapse where both OSNs send axons to the same glomerulus. We found that Glomerulus 1 OSNs had 283.4 ± 54.3 recurrent synapses per neuron, while Glomerulus 2 and 3 OSNs had only 58.9 ± 14.5 and 68.7 ± 11.1, respectively (Figs 1L and S5F). We considered the possibility that there are more recurrent synapses in Glomerulus 1 OSNs simply because their processes are longer than Glomerulus 2 or 3 OSNs (Fig 1I). Because the opportunities for neurons to be synaptically connected are limited to locations where axons and dendrites are sufficiently close to make synapses, we computed the density of recurrent synapses per micrometer of overlapping axonal cable length (when axons are within 2 µm of each other or cable overlap) in each glomerulus (Fig 1M). Glomerulus 1 OSNs had the highest recurrent synapse density with 0.053 ± 0.008 synapses per micrometer, while Glomerulus 2 OSNs had 0.021 ± 0.006 and Glomerulus 3 OSNs had 0.036 ± 0.005 (Figs 3D and S5G). To account for the difference in neurite length, we also computed the fraction of the total OSN output synapses that are recurrent synapses. We also found that Glomerulus 1 OSNs had the highest fraction of recurrent synapses (Fig 1N). The higher number of recurrent connections among Glomerulus 1 OSNs may provide a neural substrate for amplifying excitation by CO_2_ cues in the mosquito brain (Fig 1O).

**Fig 2:**
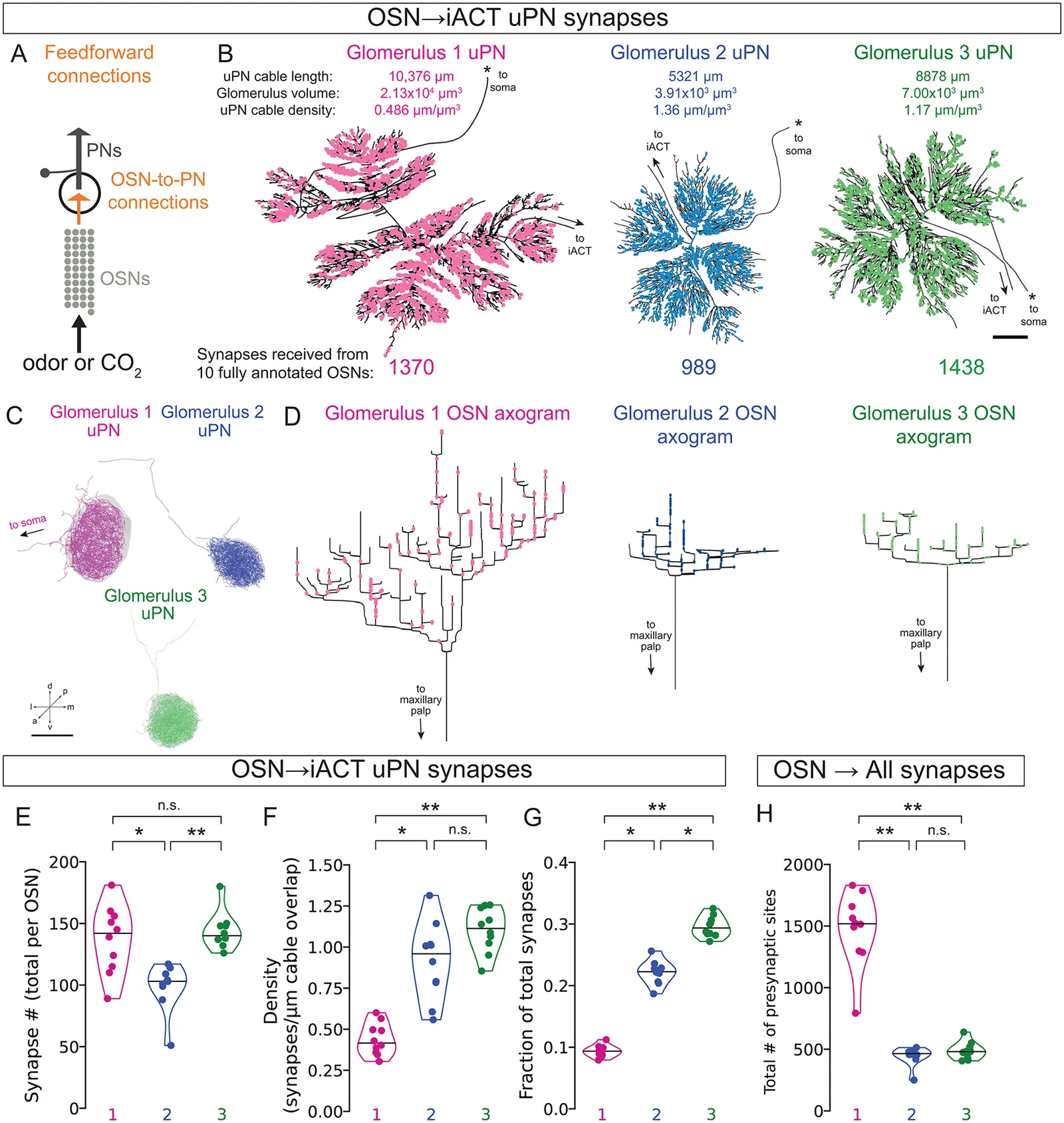
Feedforward connectivity of maxillary palp glomeruli. **(A)** Schematic with feedforward OSN-to-PN connections highlighted. **(B)** Dendrograms (flattened 2D representations of the 3D dendritic arbor preserving distance information) of uPNs and their incoming feedforward synapses from OSNs. * denotes branch leading to soma, and arrow denotes branch leading to the inner antennocerebral tract (iACT). (top) uPN cable length, glomerular volume, and cable density. Scale bar 100 µm. **(C)** Coronal view of uPN reconstructions. Scale bar 25 µm. **(D)** Axograms (flattened 2D representations of the 3D axonal arbor preserving distance information) of representative OSNs with their feedforward synapses to uPNs shown as colored dots. Scale bar 25 µm. **(E)** Number of OSN-to-uPN synapses from 10 fully reconstructed OSNs within Glomerulus 1, 2, and 3 (Kruskal–Wallis test (two-tailed), p = 0.0004, pairwise comparisons using Dunn’s post-hoc test with Bonferroni correction: n.s, *p* > 0.05; * *p* ≤ 0.05; ** *p* ≤ 0.001). **(F)** Density of OSN-to-uPN synapses per µm of cable overlap (length of uPN dendrite within 2 µm of OSN axon, see Methods) (Kruskal–Wallis test (two-tailed), *p* = 3.34 × 10^−5^, pairwise comparisons using Dunn’s post-hoc test with Bonferroni correction: n.s., *p* > 0.05; * *p* ≤ 0.01; ** *p* ≤ 0.001). **(G)** Feedforward OSN to uPN synapses as a fraction of overall OSN output synapses (Kruskal–Wallis test (two-tailed), *p* = 2.49 × 10^−6^, pairwise comparisons using Dunn’s post-hoc test with Bonferroni correction: * p ≤ 0.05, ** *p* ≤ 0.001). **(H)** Total number of outgoing OSN synapses to all cell types (Kruskal–Wallis test (two-tailed), *p* = 4.5 × 10^−5^, pairwise comparisons using Dunn’s post-hoc test with Bonferroni correction: n.s., *p* > 0.05, * *p* ≤ 0.01; ** *p* ≤ 0.001). The data underlying this Figure can be found in S5–S8 Data.

### CO_2_-sensitive OSNs have more recurrent than feedforward synapses

To study feedforward connectivity, we reconstructed antennal lobe uPNs (Fig 2A). We identified the uPNs that innervate Glomerulus 1, 2, and 3, which have large-caliber axons that project dorsally in the iACT. These were classified as iACT uPNs because of their axonal projections and because their dendrites did not extend beyond the glomerulus (S1B Fig). We found that only one ipsilateral iACT uPN innervated each glomerulus (Fig 2B and 2C). As with the OSNs, the Glomerulus 1 uPN cable length was greater, but the neurite density was lower than for Glomerulus 2 and 3 uPNs (Fig 2B).

To determine the number of feedforward synapses formed between OSNs and uPNs, we annotated all synapses onto each ipsilateral iACT uPN (Fig 2A–2C). We found that each Glomerulus 1 OSN (Figs 2D, 2E, and S5D), made 137.0 ± 26.0 (mean ± SD) synapses onto the Glomerulus 1 uPN. This uPN received 1,370 synapses from the 10 fully annotated OSNs, and therefore receives an estimated 6,000–7,000 synapses from all OSNs. Although the Glomerulus 1 OSNs had larger and more complex arbors than Glomerulus 3 OSNs (Fig 2B), Glomerulus 1 and 3 uPNs received a comparable number of synapses from each OSN, 137.0 ± 26.0 and 143.8 ± 14.0 per OSN, respectively (Fig 2E). Glomerulus 2 uPNs received the lowest number of feedforward synapses (98.9 ± 17.7). To account for the large volume of Glomerulus 1, we again calculated the feedforward connectivity as the density of feedforward synapses per micrometer of axon-to-dendrite cable (Figs 2F and S5E), the fraction of the total output synapses (Fig 2G), and the total number of OSN output synapses (Fig 2H). Glomerulus 1 OSNs had the lowest density and fraction of feedforward synapses. Taken together, the Glomerulus 1 OSNs made more recurrent synapses than feedforward synapses onto the uPN that we sampled, while Glomerulus 2 and 3 OSNs made more feedforward than recurrent synapses (Figs 1L and 2E).

### High levels of recurrent connectivity are specific to *Ae. aegypti* CO_2_ OSNs

**Fig 3:**
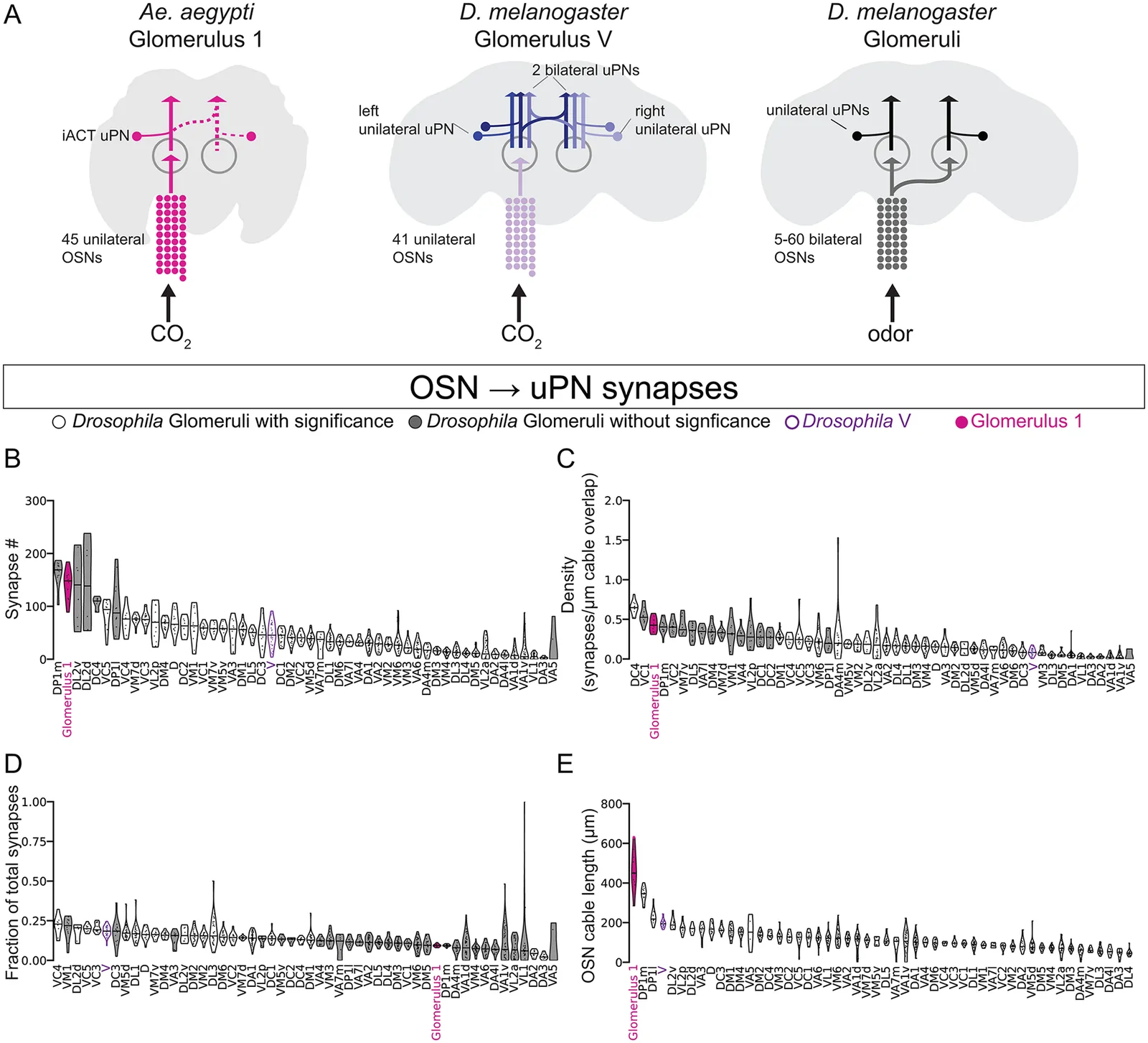
Feedforward connectivity of *Ae. aegypti* Glomerulus 1 compared to *D. melanogaster* antennal lobe glomeruli. **(A)** Schematics of cells and connectivity compared between *Ae. aegypti* and *D. melanogaster*. (left) The *Ae aegypti* CO_2_-sensitive Glomerulus 1 with 45 unilateral OSNs and the 1 uPN that projects ipsilaterally through the iACT to higher brain regions. Dashed lines indicate areas beyond the main reconstruction. (middle) The *D. melanogaster* CO_2_-sensitive glomerulus V with 41 unilateral OSNs and 3 uPNs: one unilateral projecting uPN and two bilateral projecting uPNs, with one bilateral uPN on each side of the brain. (right) Representation of *D. melanogaster* glomeruli that contain a single uPN. These contain between 5 and 60 bilateral OSNs, which each innervate the corresponding glomerulus on both sides of the brain and have 1 uPN that projects ipsilaterally to higher brain regions. **(B)** Number of feedforward OSN-to-uPN synapses for each glomerulus (Kruskal–Wallis test (two-tailed), *p* = 3.93 × 10^−56^). Filled data are non-significantly different from Glomerulus 1 using Dunn’s post-hoc test with Bonferroni correction: *p* ≤ 0.01). **(C)** Density of feedforward OSN-to-uPN synapses per µm of cable overlap within each glomerulus (Kruskal–Wallis test (two-tailed), *p* = 1.39 × 10^−45^. Filled data **N.**S. are non-significantly different using Dunn’s post-hoc test with Bonferroni correction: *p* ≤ 0.01). **(D)** Feedforward OSN-to-uPN synapses as a fraction of overall OSN output synapses (Kruskal–Wallis test (two-tailed), *p* = 4.87 × 10^−38^. Filled data are non-significantly different using Dunn’s post-hoc test with Bonferroni correction: *p* ≤ 0.01). **(E)** OSN axonal cable length within each glomerulus. (Kruskal–Wallis test (two-tailed), *p* = 1.03 × 10^−62^. Filled data are non-significantly different using Dunn’s post-hoc test with Bonferroni correction: *p* ≤ 0.01). The data underlying this Figure can be found in S1, S5–S7 Data.

**Fig 4:**
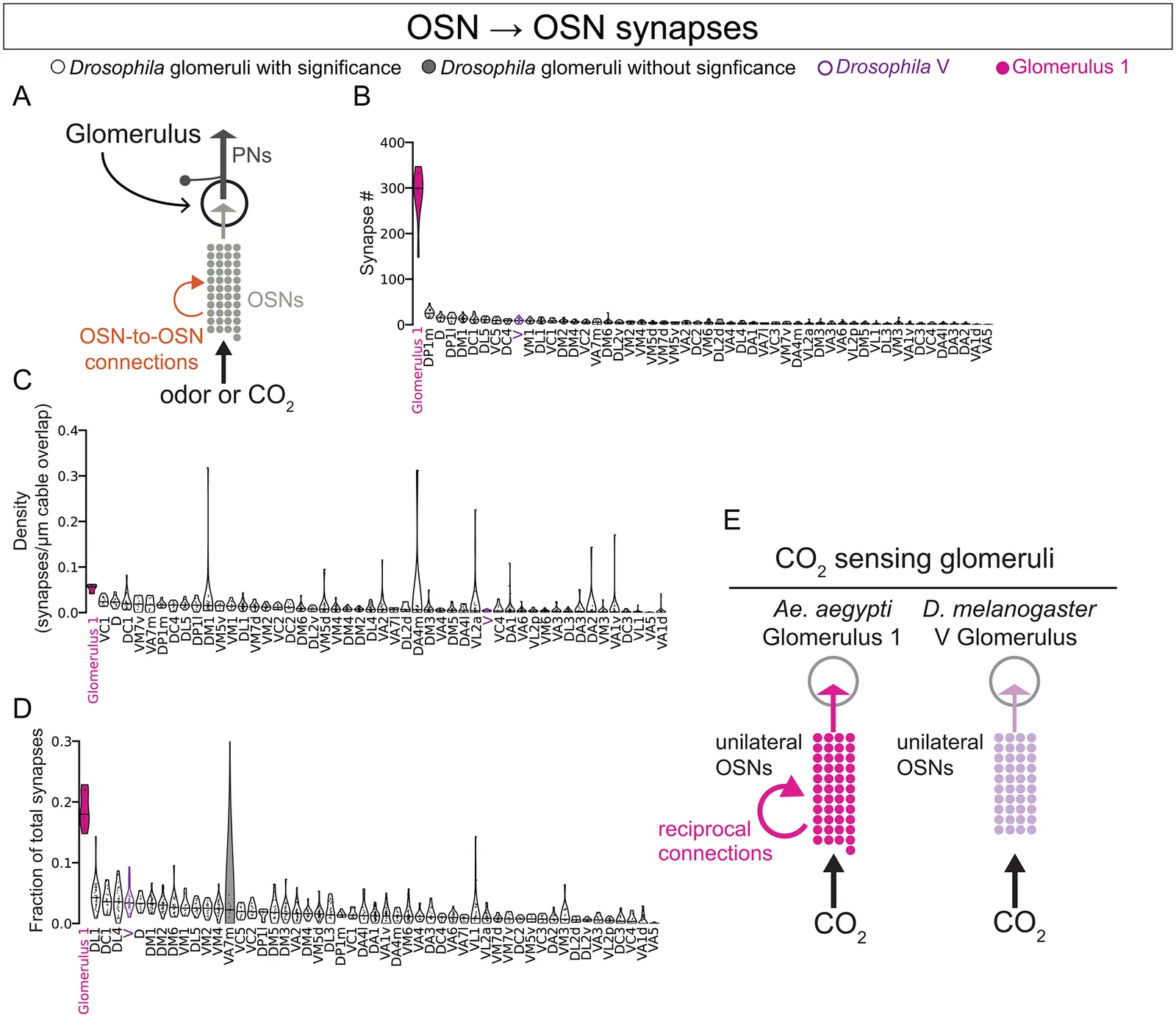
*Ae. aegypti* Glomerulus 1 has elevated reciprocal OSN-to-OSN connectivity. **(A)** Schematic with reciprocal OSN-to-OSN connections highlighted. **(B)** Total number of outgoing OSN-to-OSN synapses contained within each glomerulus (Kruskal–Wallis test (two-tailed), *p* = 1.63 × 10^−98^. Ten fully reconstructed OSNs are included for *Ae. aegypti* CO_2_-sensitive Glomerulus 1. Filled data are non-significantly different using Dunn’s post-hoc test with Bonferroni correction: *p* ≤ 0.01). **(C)** Density of reciprocal OSN-to-OSN synapses per µm of cable overlap within each glomerulus (Kruskal–Wallis test (two-tailed), *p* = 1.39 × 10^−72^. Filled data are non-significantly different using Dunn’s post-hoc test with Bonferroni correction: *p* ≤ 0.01) **(D)** Reciprocal OSN-to-OSN synapses as a fraction of overall OSN output synapses (Kruskal–Wallis test (two-tailed), *p* = 1.13 × 10^−72^. Filled data are non-signficantly different using Dunn’s post-hoc test with Bonferroni correction: *p* ≤ 0.01). **(E)** Schematic summarizing difference between CO_2_-selective OSN connectivity between *Ae. aegypti* and *D. melanogaster*. The data underlying this Figure can be found in S2–S4 Data.

While CO_2_ initiates an activated search state in female *Ae. aegypti* mosquitoes, it has a different impact on *D. melanogaster* behavior. For *D. melanogaster*, CO_2_ is aversive when the fly is at rest, and CO_2_ can be attractive when flies are already in motion [60–64]. Although most of the olfactory systems of both insects utilize odorant receptors (ORs) and ionotropic receptors (IRs), CO_2_ is detected by a gustatory receptor (GR) that is composed of Gr1, Gr2, and Gr3 subunits in *Ae. aegypti*, which are orthologues of Gr21a and Gr63a subunits in *D. melanogaster* [65,66]. We sought to determine if the organization of the CO_2_ glomerulus is dictated by the receptor expressed in its OSNs or if ethological significance is more predictive of circuit structure. To this end, we compared circuit features of the *Ae. aegypti* CO_2_ glomerulus to all *D. melanogaster* olfactory glomeruli, including the CO_2_ glomerulus V (Figs 3 and 4).

We first compared feedforward synaptic connections between *Ae. aegypti* Glomerulus 1, and all *D. melanogaster* glomeruli. We leveraged our *Ae. aegypti* dataset and the *D. melanogaster* Full Adult Fly Brain dataset [30] using segmentations and synapse predictions available in FlyWire [28,67]. OSN projections to all *Ae. aegypti* glomeruli are unilateral, as are OSN projections to *D. melanogaster* glomerulus V. All other *D. melanogaster* olfactory glomeruli receive feedforward input from bilateral OSNs (Figs 3A, S6, and S7). To account for these morphological differences, we restricted our analysis to neurites and synapses within each right glomerulus in FlyWire. To compare feedforward connectivity across these glomeruli, we quantified the number of synapses each OSN makes onto its cognate uPNs (Fig 3B). We found that the feedforward synapse number (Fig 3B), feedforward synapse density (Fig 3C). and feedforward fraction of synapses (Fig 3D) of Glomerulus 1 were similar to many of the fly glomeruli, despite the large size of this glomerulus, however, the neurite length was greater than any of the fly glomeruli (Fig 3E). The total number of feedforward synapses in Glomerulus 1 was on the high end of the distribution, but was not significantly different from some of the fly glomeruli, including DC4 and DP1m, which have both been implicated in acid sensing [68,69].

The *D. melanogaster* V glomerulus is innervated by dendrites from a unilateral uPN in each hemisphere, and two bilateral uPNs, one with a soma in the left hemisphere and one in the right hemisphere (Fig 3A). In order to compare feedforward OSN-to-uPN connectivity between Glomerulus 1 and the fly V glomerulus, we included the three uPNs that innervate the right V glomerulus in the analysis: the right uPN, right bilateral uPN, and left bilateral uPN (S9A–S9C Fig). In glomerulus V, the unilateral uPN received 5.4 ± 4.0 synapses from each OSN, and the right and left bilateral uPNs received 22.4 ± 10.0 and 19.9 ± 10.3, respectively (S9B Fig). The total number of feedforward synapses was 47.7 ± 22.1, which was lower in number (Fig 3B) and density (Fig 3C) than Glomerulus 1. The total fraction of feedforward synapses in V was higher than in Glomerulus 1 (Fig 3D). In summary, although Glomerulus 1 and V respond to the same sensory cue, the feedforward connectivity of Glomerulus 1 does not resemble V relative to other fly olfactory glomeruli.

We next compared recurrent OSN connectivity in *D. melanogaster* glomeruli to Glomerulus 1 (Fig 4A). We found that Glomerulus 1 OSNs made ~11-times more recurrent synapses (283.4 ± 53.4) than OSNs of any fly glomeruli (Fig 4B), and Glomerulus 1 had elevated OSN-to-OSN synapse density (Fig 4C) and fraction (Fig 4D) compared to *D. melanogaster* glomeruli. V was not similar to Glomerulus 1 in any of these metrics. We also found that all three *Ae. aegypti* maxillary palp glomeruli had higher numbers of recurrent connections than the fly glomeruli (S8 Fig), although we note that none compared to the high levels seen in Glomerulus 1. Based on snRNA-seq expression data [70], these OSNs are cholinergic, and the CO_2_-sensing CpA neurons express orthologues of four of the nicotinic acetylcholine receptor subunits (S9D and S9E Fig and S1 Table), suggesting these are excitatory connections. Taken together, Glomerulus 1 has comparable levels of feedforward connectivity, but far more recurrent connectivity than olfactory glomeruli in *D. melanogaster,* including the CO_2_-sensing V glomerulus (Fig 4E).

### Putative ribbon-like synapses are specifically between *Ae. aegypti* CO_2_ sensory neurons

**Fig 5:**
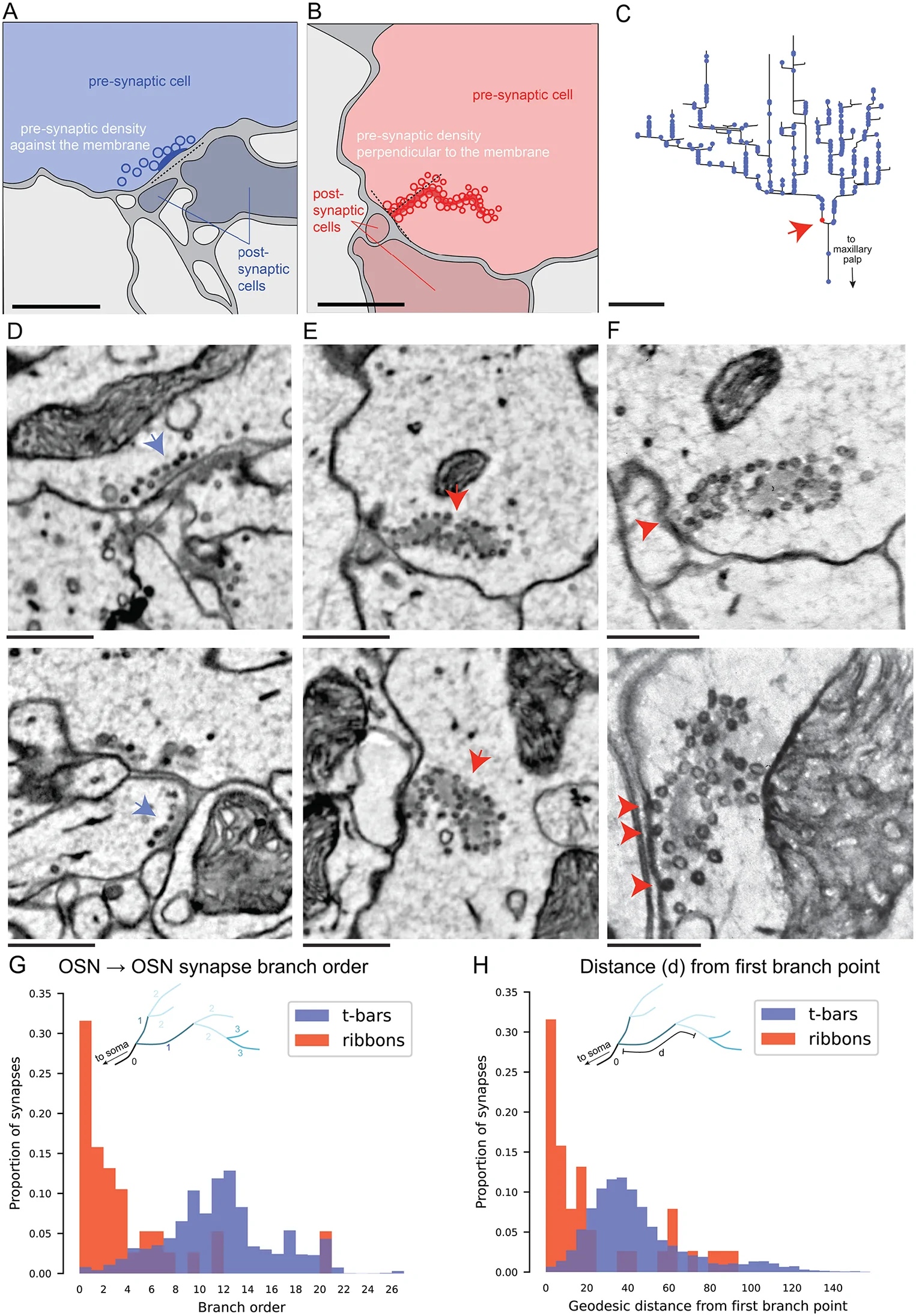
Ribbon-like synapses among CO_2_ OSNs. **(A)** Schematic of a canonical T-bar synapse and **(B)** a putative ribbon-like synapse in *Ae. aegypti* Glomerulus 1. Scale bars 500 nm. **(C)** Axogram of an example Glomerulus 1 OSN with reciprocal output T-bar (blue) and putative ribbon-like (red) synapses represented by colored dots. Arrow points to a putative ribbon-like synapse from this OSN. Scale bar 25 µm. **(D)** EM micrographs of canonical T-bar (blue arrows) synapses. Scale bars 500 nm. **(E)** EM micrographs of putative ribbon-like (red arrows) synapses. Scale bars 500 nm. **(F)** Higher magnification (0.5 nm/pixel) EM micrographs of putative ribbon-like synapses with vesicles docked to the presynaptic membrane (arrowheads). Scale bars 200 nm. **(G)** Histogram of putative ribbon-like and T-bar OSN-to-OSN synapse branch order (Mann–Whitney *U* test, *p* = 5.93 × 10^−17^). Inset: illustration of synapse branch order. **(H)** Histogram of distances (cable length) from the first branch point to putative ribbon-like and T-bar OSN-to-OSN synapses (Mann–Whitney *U* test, *p* = 6.78 × 10^−9^). Inset: illustration of quantified distance (geodesic distance or cable length) of synapse from the first branch point. The data underlying this Figure can be found in S9 and S10 Data.

Synapses in *D. melanogaster*, and most other insects are typified by presynaptic structures called T-bars, named for their recognizable “T” shaped presynaptic density when viewed in cross-section [45,47]. We were surprised to identify two morphologically distinct categories of electron-dense specializations in *Ae. aegypti*. The majority of presynaptic structures resembled T-bars in the fruit fly (Fig 5A and 5D), although in *Ae. aegypti*, the base or “pedestal” of the T-bars appears shorter in our specimen. The “table-tops” of the T-bars are oriented parallel to the presynaptic membrane and are lined with synaptic vesicles on the side distal to the membrane. T-bars make up the vast majority of synapses in our dataset and occur between all combinations of cell types we examined.

We also found a number of electron-dense structures that were oriented perpendicularly to the cell membrane and resemble ribbon synapses found in mammalian retina and vertebrate hair cells, which are named for their elongated ribbon-like morphology (Figs 5B, 5E, S10, and S11 and S2 Table) [44,46,51–53]. These densities were lined with vesicles on both sides. These putative ribbon-like synapses were only seen in OSNs that innervate Glomerulus 1 and are only located at recurrent contacts between CO_2_-sensitive OSNs. Within the reconstructed OSNs, we saw a total of 60 ribbon-like specializations, around 30 on each side of the brain. These ribbon-like structures are located most often near the primary branches of OSNs close to the point where the axon first enters the glomerulus, while T-bars are found throughout the entire OSN arbor in the glomerulus (Fig 5C, 5G, and 5H). We did not detect ribbon-like structures in OSNs or PNs in any other glomeruli we analyzed, in neither *Ae. aegypti* nor *D. melanogaster*, suggesting they may be specific to CO_2_-sensitive Glomerulus 1 OSNs in the mosquito.

To determine whether these ribbon-like structures could be presynaptic specializations that participate in synaptic transmission, we looked for the presence of vesicles that were “docked” at the presynaptic membrane. Docked vesicles contact the presynaptic membrane in preparation for rapid exocytosis to release neurotransmitter into the synaptic cleft [41]. To identify putative docked vesicles, we re-imaged 19 ribbon-like structures at higher magnification (0.32 × 0.32 nm^2^ per pixel and 0.4 × 0.4 nm^2^ per pixel). We identified instances where vesicles were in contact with the presynaptic plasma membrane (Fig 5F), which is a signature of docked vesicles. This supports the hypothesis that these ribbon-like structures may function as presynaptic specializations involved in synaptic transmission.

### Modeling reveals a role for recurrent connections in enhancing CO_2_ detection

**Fig 6:**
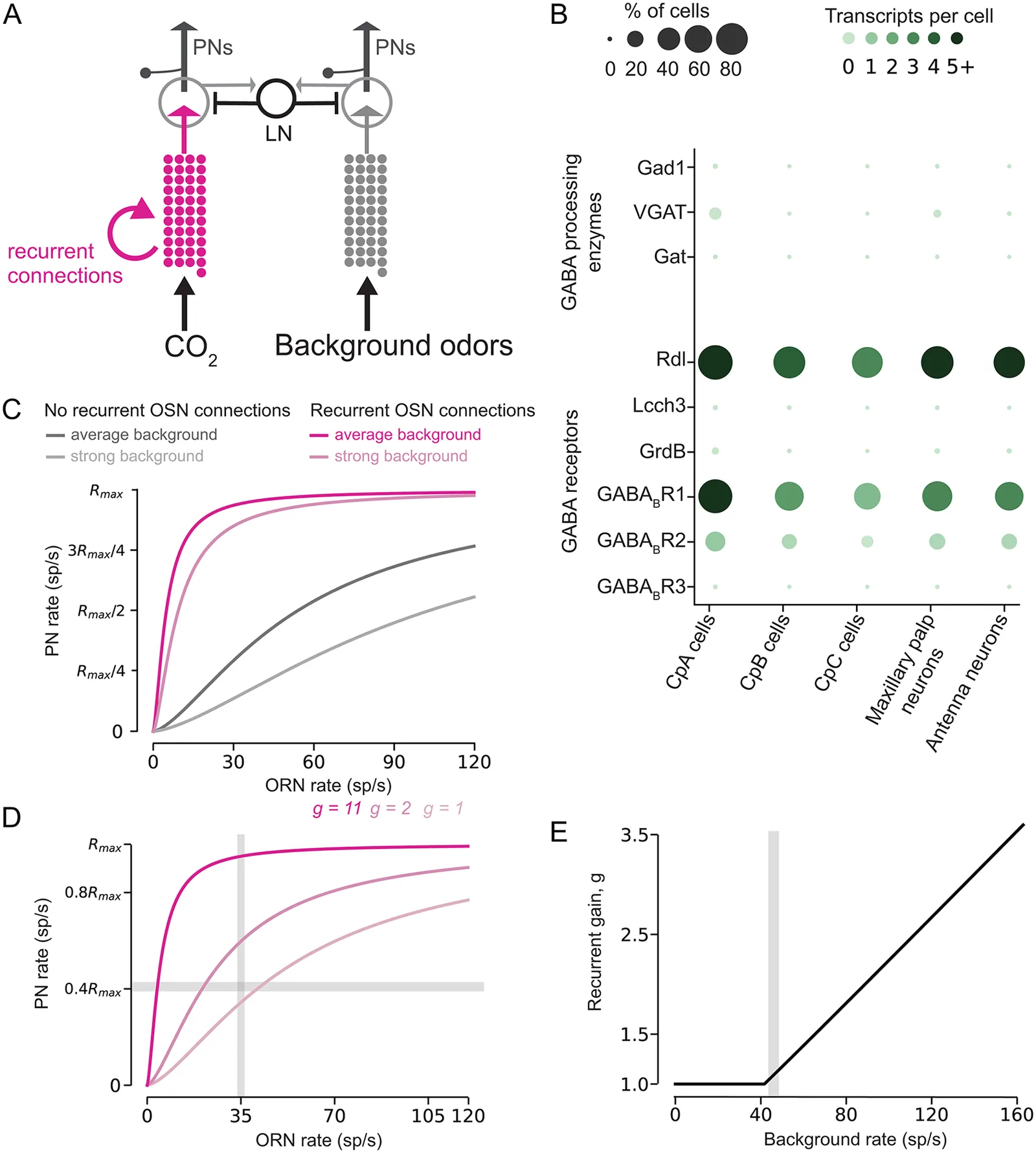
Connectomically-informed network model reveals recurrent connectivity specifically enhances stimulus detection. **(A)** Model architecture. CO_2_ is processed by Glomerulus 1, which includes recurrent connections. Co-occurring odorants (‘background’) activate glomeruli which inhibit other glomeruli through inhibitory local neurons (LNs). (B) snRNAseq data replotted from ref [70]. **(C)** OSN to PN response curves for OSNs with and without recurrent connections, for two different background odor strengths (average and strong, see Methods). **(D)** OSN to PN response curve for differing values of *g* for an average background odor strength. Vertical gray shaded region indicates OSN response rates for which CO_2_ should be detected (35 sp/s), and horizontal gray shaded region indicates the PN response strength that would correspond to CO_2_ detection (0.4*R*_*max*_). **(E)** Minimum value of *g* required to ensure CO_2_ detection for varying background odor strengths. Vertical gray shaded region indicates an average level of background odor.


To better understand the impact of recurrent connections on olfactory encoding, we constructed a mathematical model that captured key elements of olfactory processing (Fig 6A). We envisioned an otherwise standard *D. melanogaster* olfactory system, with a single specialized CO_2_ glomerulus with excitatory recurrent connections. Following existing approaches [71,72], we modeled the OSN-to-PN circuit including inhibitory gain normalization. In *D. melanogaster*, inhibitory local neurons (LNs) in the antennal lobe mediate interglomerular inhibition upon odorant activation (Fig 6A). GABA receptors are expressed in OSNs, including the CO_2_-sensing CpA neurons, consistent with the presence of lateral inhibition by LNs in the *Ae. aegypti* antennal lobe (Fig 6B and S1 Table). We considered how LN inhibition impacts Glomerulus 1 PN activity when other glomeruli, which lack recurrent connections, are activated by background odorants, thereby increasing LN activity. This model took the following form:

$$\begin{array}{c} r_{PN}=R_{max}\left(\frac{r_{ORN}^{\text{1}.\text{5}}}{r_{ORN}^{\text{1}.\text{5}}+s^{\text{1}.\text{5}}+\sigma^{\text{1}.\text{5}}}\right) \end{array}$$

where *R*_*max*_ is the maximum PN response rate, *s* mediates inter-glomeruli divisive inhibition via a weighted average of all glomeruli to a given odorant, and σ defines the OSN value that achieves half-max activity. Values of σ, *R*_*max*_, and the exponent of 1.5 were adopted from [71]. Recurrent connections were incorporated as a multiplicative gain *g* applied to OSN activity $\left(r_{ORN}\to {g\cdot r}_{ORN}\right)$. We viewed this as the collective gain increase due to anatomical connections as well as their effective strength, which we estimated from EM data. We simulated our model under different scenarios to illustrate the impact of recurrent OSN connections on PN activity. To simulate CO_2_ in the presence of other odors (‘background odors’), we set *s* = 50 spikes/s, consistent with previous models [71–73]. We refer to background odors of this strength as ‘average’ background.

We simulated PN responses to different OSN levels when recurrent connections were present (Fig 6C, magenta) and were not present (Fig 6C, gray). Recurrent connections were simulated by increasing *g* by a factor of 11, simulating the presence of 11-times the number of recurrent connections. Recurrent connections led to a sharp increase in the PN response curve, yielding a significantly larger PN response to smaller OSN activity. To examine the sensitivity of this gain to increases in the background odor, we doubled *s* to 100 spikes/s (Fig 6C, ‘strong background’), demonstrating that global OSN activity decreases individual PN response due to lateral inhibition. These results are consistent with a hypothesis that recurrent connections amplify weak OSN input for the important ethological cue of CO_2_.

Our primary hypothesis is that recurrent connections in Glomerulus 1 facilitate CO_2_ detection. To illustrate this, we considered a detection task where a PN response greater than a threshold *R*_*thresh*_ = 0.4*R*_*max*_ indicated the presence of CO_2_ (Fig 6D). For accurate CO_2_ detection, PN activity should reach *R*_*thresh*_ for CO_2_ concentrations found to elicit weak OSN responses. Based on published results [74], we identified 35 sp/s as the OSN threshold for CO_2_ response detection, for which PNs should become robustly active.

We explored values of *g* that achieved a PN response greater than or equal to *R*_*thresh*_ for OSN responses equal to 35 sp/s, for an average background odor (Fig 6D; *s =* 50). We also identified the minimum value of *g* that achieved this for a range of background odor strengths (Fig 6E). For average background odor strength, both *g* = 11 and *g* = 2 (Fig 6D, dark and medium magenta, respectively) produced responses greater than 0.4*R*_*max*_ for OSN values of 35 sp/s, while *g* = 1 (Fig 6D, light magenta) did not. We found that for average or stronger background odor strengths (*s* = 50 or greater) *g* needed to be greater than one to achieve detection of transient CO_2_ increases (Fig 6E), indicating that recurrent connections are necessary even under normal environmental conditions to ensure robust detection. Empirically, we measured a relatively large value for *g* that may suggest: (1) an extremely strong preference for detecting low levels of CO_2_, even at the expense of false alarms; (2) inhibition is significantly stronger than excitation in this circuit, and/or (3) the recurrent synapses we identified are effectively weak despite their number. Taken together, our modeling results demonstrate how recurrent connections between OSNs can amplify weak sensory responses to achieve robust stimulus detection even in the presence of realistic background odor levels.

## Discussion

To investigate the organizational principles of neuronal networks supporting innate sensory-driven behavior, we present the first central microcircuit connectome in the *Ae. aegypti* mosquito. We used automated tape-based transmission EM [18] and manual reconstruction to trace and annotate the wiring and connectivity of all three sensory glomeruli innervated by the maxillary palp, including the CO_2_-sensitive glomerulus (Glomerulus 1). We found that each glomerulus had an array of 45–46 OSN inputs, and that the OSNs that innervate the CO_2_-responsive Glomerulus 1 had a uniquely high level of recurrent OSN-to-OSN connectivity. Among the recurrent connections were structures that resemble ribbon synapses, which may couple OSNs to one another. We identified a single uPN that projects through the ipsilateral iACT in each palp glomerulus and analyzed feedforward connectivity onto these neurons. Using comparative connectomics, we found that the feedforward wiring of the *Ae. aegypti* CO_2_-sensitive glomerulus is similar to glomeruli in *D. melanogaster*, but with far higher recurrent connectivity. We found no obvious similarities between Glomerulus 1 and the V glomerulus, which share related receptors for CO_2_. Our results suggest that highly recurrent excitatory connectivity between CO_2_-sensitive OSNs in *Ae. aegypti* may amplify CO_2_ detection to enable behavioral activation and sensitization. We further suggest that similar features of behavioral salience may predict the architecture of microcircuits across species. In support of our experimental findings, we demonstrated with network modeling how recurrent connections improve stimulus detection under normal environmental conditions and in the face of strong background odors which might make detection harder.

### From the periphery to the brain

Recent work used serial block-face scanning EM to characterize the morphology of the dendrites, cell bodies, and axons of same OSN types in the periphery, at the level of the sensory organ [75]. The CO_2_-sensing neuron, often referred to as CpA (capitate peg sensillum A), and the two odor-sensitive neurons, CpB and CpC (capitate peg sensillum B and C), are all housed in the basiconic (also called capitate peg) sensilla of the maxillary palp. A recent study identified flattened dendritic sheets that fold into elaborate lamellae, creating a major expansion in the dendritic surface area only in the CO_2_-sensing CpA dendrites [75]. Our work complements this anatomical report of the dendrites with an analysis of the synaptic organization of their axons in the brain. Both studies highlight differences between CO_2_-sensing neurons in *Ae. aegypti* and odorant-sensing neurons and together these findings suggest that in *Ae. aegypti*, CO_2_ is uniquely processed as a sensory cue consistent with its potent role in behavior.

### Recurrent excitatory connectivity

What is the functional role of recurrent synapses between OSNs? We predict that excitatory recurrent connections between Glomerulus 1 OSNs could support signal amplification, propagation, persistent activity, or a combination of these processes. Recurrent amplification has been reported in multiple sensory systems. In the mammalian sensory cortex, excitatory pyramidal neuron responses are thought to arise from selective amplification of thalamocortical signals [76–78] through recurrent connections between similarly selective excitatory neurons [59,79–82]. In *D. melanogaster*, a recurrent connectivity motif supports sustained activity for olfactory learning in the mushroom body [83]. It may be surprising that amplification would occur so early in sensory processing; however, CO_2_ is a particularly important cue for *Ae. aegypti*, and the location of these recurrent connections would provide a mechanism to amplify CO_2_ signaling prior to inhibition by LNs. This may create a mechanism that could surmount the strong and broad inhibitory tone provided by LNs in the presence of other chemosensory input [84]. By amplifying weak inputs, recurrent connections lower the effective detection threshold, which increases sensitivity, but also increases the risk of false alarms from noise or transient fluctuations in OSN activity. This is a classic example of a sensitivity-specificity trade-off and may reflect a circuit-level prioritization of detection over discrimination for CO_2_ in *Ae. aegypti*.

Reliable signal propagation across long distances is a general challenge of nervous systems [85]. Notably, the reconstructed OSNs and their recurrent connections are far from their sensory endings in the maxillary palp. Given that these unmyelinated axons travel a long distance along the maxillary palp nerve and subsequently branch and ramify as they enter the glomerulus, one possibility is that recurrent synapses between OSNs may provide a mechanism to mitigate signal propagation failures and increase the robustness of CO_2_ detection. Consistent with this idea, recurrent connections in the mammalian cortex enhance the reliability of sensory signals for subnetworks with more recurrent connectivity, and loss of a few neurons in the recurrent network results in degraded stimulus encoding [81].

Recurrent connectivity has been widely associated with sustained and persistent activity, a hallmark of short-term working memory [86,87]. In the mammalian cortex, recurrent connections are hypothesized to facilitate this process, providing a neural substrate for maintaining information over short timescales. However, examples of recurrence extend beyond the cortex. In invertebrates, one notable case is the sustained bump of activity in the ring attractor networks of the *D. melanogaster* central complex, which plays a key role in spatial navigation [88,89]. Persistent activity in the CO_2_ sensory system is an intriguing possibility in female *Ae. aegypti,* where CO_2_ elicits a prolonged activated behavioral state. CO_2_ sensitizes the organism and, when integrated with other host cues, drives innate host-seeking behavior that typically lasts minutes [90]. Notably, Glomerulus 3 OSNs respond to the host odorant 1-octen-3-ol and also have recurrent OSN-to-OSN connections, although fewer than between CO_2_-sensitive OSNs, while Glomerulus 2 OSNs have relatively few recurrent connections and are not clearly characterized as host-detecting neurons [39]. The role of recurrent connections in host detection and neuron physiology remains to be seen, as virtually all mosquito OSN electrophysiology has been performed at the sensory periphery, far from the recurrent connections we describe [91].

A recent analysis of a *D. melanogaster* hemibrain connectome [27] reported the presence of recurrent axo-axonic connections between OSNs in *D. melanogaster* [92]. Analysis of this connectome diverged from our findings using the FlyWire connectome. In this work, network modeling demonstrated how activity-dependent recurrent connections for every glomerulus could lead to greater odor discrimination by pattern decorrelation, effectively supporting the functional role long established for divisive inhibition [71,72]. Our work demonstrates an alternative, but complementary role for recurrent connections for a single, high-priority glomerulus: odor detection via signal amplification. Further experimental investigation is necessary to understand how species-specific olfactory systems balance these two computations, perhaps as a function of ethological niche.

### Invertebrate ribbon-like synapses

We identified structures specifically between CO_2_-sensitive OSNs that resemble ribbon synapses seen in vertebrates. Examples of vertebrate ribbon synapses include retinal photoreceptors and bipolar cells [53,93], auditory hair cells [51], and fish lateral line hair cells [52], where ribbon synapses scaffold vesicle pools that enable sustained, graded release [44,46] (S10 Fig). Presynaptic ribbon-like structures have not previously been reported in insects and have not been reported in recently generated *D. melanogaster* connectomes. If these structures are indeed ribbon synapses, they would be the first identified in invertebrates, although we note that their function in neurotransmission remains to be studied.

Vertebrate ribbon synapses contain a protein encoded by the *ribeye* gene [50], which makes up the electron-dense structure of the ribbon [50,94,95]. The Ribeye protein contains two domains: the A domain that is believed to confer ribbon structure at active zones, and the B domain that is orthologous to the transcriptional repressor with enzymatic activity, CtBP2 [46,50]. Although there are invertebrate gene orthologues of the *CtBP2* B domain, there are no orthologues of the *ribeye* A domain that is required for ribbon assembly. The absence of the *ribeye* domain from invertebrate genomes has led to the assertion that ribbon synapses are a vertebrate specialization. We speculate that the development of ribbon-like synapses in vertebrates and invertebrates may be a case of convergent evolution. Alternatively, invertebrates may have a Ribeye-like scaffold protein encoded by a divergent DNA sequence that hinders detection with DNA alignment algorithms, but nonetheless has a similar protein structure. Further study is needed to investigate the proteins that compose these structures.

We observe ribbon-like structures exclusively at recurrent contacts between CO_2_-sensitive OSNs and only at the most proximal axon branches, which correspond to the entry point of axons into Glomerulus 1. The locations of these putative ribbon-like synapses suggest that they may amplify recurrent connectivity specifically among CO_2_ sensory neurons.

Finally, it remains possible that these are not ribbon synapses, but rather a structure that appears similar when imaged with transmission EM. It is a challenge to explore their function because they are located deep in the antennal lobe, they are in small axo-axonic connections, and we currently lack an identified protein component such as Ribeye to gain genetic access to these structures. Given their close proximity with synaptic vesicles, one alternative is that they are specializations involved in trafficking or otherwise organizing vesicles in CO_2_-selective OSNs. It is notable that these specializations have, to our knowledge, not been previously reported in insects and appear specifically in *Ae. aegypti* OSNs selective for CO_2_. Future work is needed to uncover their functional role in mosquito CO_2_ processing.

### Circuit logic is not based on receptor alone

*Ae. aegypti* females are obligate blood drinkers, while *D. melanogaster* are dietary generalists that feed on decaying plant matter. The two Dipteran species diverged over 150 million years ago [37,38] and retain the same gross brain morphology [54,96]. Here, we compared the connectivity of the olfactory system of these divergent feeders at synaptic resolution. We observed a number of similarities between both organisms, including the prevalence of T-bar-like synapses as the predominant presynaptic specialization, although we note slight differences in the morphology of the T-bars. Some additional differences were clear at the onset of this study. *Ae. aegypti* OSNs send unilateral projections to the ipsilateral glomerulus while those in *D. melanogaster* largely send bilateral projections to mirror symmetric glomeruli in both the ipsi- and contralateral antennal lobes. One exception to this rule is the CO_2_-sensitive OSN projections to the *D. melanogaster* V glomerulus, which are exclusively ipsilateral. It is notable that CO_2_-sensitive OSNs in *Ae. aegypti* are housed in the maxillary palp, a different sensory organ than their location in the *D. melanogaster* antenna.

The role of CO_2_ differs between these species. In *D. melanogaster*, behavioral response to CO_2_ is context-dependent and can be either aversive or attractive [60–64]. Flies avoid regions of high CO_2_ concentration, which is a signature of inhospitable environments [63]. CO_2_ is also a component of an odorant mixture that *D. melanogaster* emits when stressed, and conspecifics innately avoid this mixture [61]. However, CO_2_ can also be attractive, as decaying food sources release this cue [60,64]. While *D. melanogaster* is attracted to CO_2_ during active foraging, attraction relies on a separate receptor and circuit that utilizes the subunit Ir25a [64]. The feedforward connectivity of CO_2_ neurons in *Ae. aegypti* Glomerulus 1 and *D. melanogaster* glomerulus V at the first synaptic relay in the brain were not particularly similar compared to all other glomeruli in *D. melanogaster*. An underlying relationship between this sensory architecture and the valence or salience of sensory cues may exist, but we did not see a clear correlation. However, the recurrent connectivity of CO_2_-sensitive OSNs was strikingly different, suggesting differential signal processing between species.

### Beyond CO_2_ sensitization: Host cue integration in the *Ae. aegypti* brain

Effective host-seeking behavior requires integration of multiple sensory cues [5–10,12,39]. Our analysis of the CO_2_ system lays the groundwork for studying the integration of CO_2_ with other host cues in the *Ae. aegypti* brain. The boundaries of the EM volume analyzed here did not allow for the analysis of the complete CO_2_ circuit. There are likely additional uPNs and MGs that innervate these glomeruli, including a network of LNs. Our feedforward analysis is restricted to only part of the circuit, and future work to describe the entire CO_2_ pathway will be a major step towards understating how multiple cues are integrated in the mosquito brain. In *D. melanogaster*, PNs mainly project to two higher-order brain areas: the mushroom body, crucial for learning and memory, and the lateral horn, thought to be critical for sensory-driven innate behavior [97–99]. As Glomerulus 1 is innervated by an ipsilateral iACT-projecting uPN, we predict that multiple sensory pathways converge in the mosquito lateral horn to support innate host-seeking behavior. Examining such hypotheses awaits an *Ae. aegypti* whole-brain connectome.

## Methods

### Experimental model and subject details

*Aedes aegypti* wild-type laboratory Liverpool mosquitoes (LVPib12 strain) were maintained and reared at 25–28 °C, 70%–80% relative humidity, with a photoperiod of 14 hr light:10 hr dark as previously described [100]. Adult mosquitoes were provided constant access to 10% sucrose. Male and female mosquitoes were housed together. The specimen used for this EM dataset was an adult female that was dissected nine days post-eclosion.

### Specimen preparation

Procedures involving animals were conducted in compliance with NIH guidelines and approved by the IACUC at Rockefeller University and Harvard Medical School. We fixed and stained the brain of one adult female *Aedes aegypti*. After the mosquito was immobilized on ice, the head was removed and fixed for three hours in 2.5% paraformaldehyde and 2.5% glutaraldehyde in cacodylate buffer (0.1 M cacodylate buffer at pH 7.4 with 0.04% CaCl_2_). The brain was dissected out of the head capsule in ice-cold phosphate-buffered saline (PBS) and stored in fresh fixative overnight.

The dissected brain was then washed three times for 10 min each with 0.02 M 3-amino-1,2,4-triazole (A-TRA) [30,101] in cacodylate buffer, stained in 1% osmium tetroxide, 0.1 M A-TRA in cacodylate buffer for 90 min on ice, washed in cold cacodylate buffer, and brought up to room temperature. Following a second round of staining in 2% OsO_4_ (aqueous), the specimen was rinsed in filtered double distilled water (ddH_2_O), placed in 1% thiocarbohydrazide (aqueous) at 40 °C for 8 min, then rinsed in ddH_2_O again. The brain was rinsed in maleate buffer (pH 5.15) and stained in 1% uranyl acetate in maleate buffer overnight at 4 °C.

The following day, the brain was washed in maleate buffer (pH 5.15) followed by ddH_2_O. It was stained in lead aspartate for 3 hours at 60 °C and washed in ddH_2_O. The brain was dehydrated in a graded ethanol series on ice, infiltrated, and embedded in resin (LX-112, Ladd Research). The embedded brain was polymerized at 60 °C for ~48 hours.

### Automated ultramicrotomy

The embedded specimen was automatically serially sectioned onto a tape-based collection substrate as described previously [18]. Briefly, the resin block with the brain was trimmed (Trim 90 diamond knife, Diatome) into an oblong hexagonal shape. The block was sectioned onto a Kapton substrate, GridTape (Luxel Corp), along the anterior–posterior axis using a customized an automated tape-collecting microtome (ATUM) [18,102] system attached to an ultramicrotome (Leica UC7). We targeted 40 nm section thickness. The automatically collected sections were accurately positioned (within approximately 200 µm from slot to slot) onto GridTape slots.

### Annotation of regions of interest

We used a custom-made tape-handling machine with in-line illumination and a reel-to-reel system to capture overview images of slots and annotate regions of interest (ROIs) containing the brain. We specified regions of interest for EM imaging using a custom MATLAB script.

### Automated transmission EM imaging

The GridTape reel containing the serial sections was placed into a custom reel-to-reel sample stage for continuous automatic acquisition [18] on a transmission EM (JEOL 1200 EX) with a 2 × 2 array of sCMOS cameras (Zyla 4.2, Andor). Magnification was 2,500× on the microscope, accelerating potential was 120 kV, and the beam current was ~90 microamperes through a tungsten filament. Using the annotated ROIs, micrographs of 1,747 sections spanning the posterior antennal lobes and including the three palp glomeruli were acquired at a resolution of 4 × 4 nm per pixel.

Across the series of 1,747 imaged sections comprising the dataset, there were 29 single-section losses, with no consecutive section losses. Staining artifacts were usually small and did not adversely affect annotation, especially in large neuronal processes. Small, <1 µm membrane breaks occurred in some large caliber axonal processes, but were straightforward to overcome by trained annotators.

For the posterior 746 sections, ROIs were not annotated prior to imaging. Instead, taking advantage of consistent section placement, we created a “blind” ROI that was ~1.75× the width of the previously placed ROIs, which added imaging time but captured all the tissue. This approach yielded the same results as placing smaller ROIs prior to imaging.

### Image data alignment

Raw images were aligned using a custom pipeline based on AlignTK (https://mmbios.pitt.edu/aligntk-home) on the O2 computing cluster at Harvard Medical School as described previously [18,24,103,104]. First, camera image tiles were stitched into continuous two-dimensional montages, and then consecutive montages were aligned into a three-dimensional volume. Every 50th section was used as a global constraint on the full dataset’s alignment. For sections with small artifacts that would warp or misalign with the standard elastic alignment, rigid constraints were applied (absolute_maps in AlignTK), and manually placed control points were used to map corresponding features in neighboring sections.

### Ribbon synapse manual imaging at 25,000× magnification

We performed higher-resolution re-imaging to examine the ultrastructure of ribbon synapses in greater detail. We targeted 18 out of 57 ribbons, which appeared to exhibit vesicle docking to the presynaptic membrane. Following 25 months in a dessicator (Secador, EMS), the reel was reloaded into the microscope. Single camera images at 25,000× were captured manually. Background was subtracted (rolling ball radius 50 pixels, light background) and local contrast was enhanced (histogram equalization, saturated pixels 0.4%) in ImageJ (version 1.52a) at a resolution of 0.4 × 0.4 nm per pixel.

### Anatomical nomenclature

Throughout this work, we use the nomenclature proposed for glomeruli in [39]. We refer to the maxillary palp glomeruli as Glomerulus 1, 2, and 3. These glomeruli are referred to in previous literature as medio-dorsal (MD) glomeruli MD1, MD2, and MD3, respectively [54,105]. The glomeruli in *Ae. aegypti* were originally named [54] using a set of coordinate axes that is different from what is now commonly used in *D. melanogaster* [106], and these glomeruli are not notably dorsal in the coordinate space that we use here. We have therefore chosen the naming scheme that refers to them as Glomerulus 1, 2, and 3 (see [39] for a more complete description of antennal lobe glomeruli nomenclature).

### Reconstruction of *Ae. aegypti* neurons

Neurons were manually reconstructed in the EM dataset as described previously [18,24,79]. We imported the data into Collaborative Annotation Toolkit for Massive Amounts of Image Data (CATMAID, release 2018.11.09) [107] for distributed web-based reconstruction and annotation. To reconstruct neurons, we manually placed points, or nodes, down the center of each process, creating skeletonized models of neuron morphology.

We identified the maxillary palp nerve bundle on the right side of the brain, with corroboration from light-level images, then completed tracing of 45 Glomerulus 1 OSNs, 45 Glomerulus 2 OSNs, and 46 Glomerulus 3 OSNs with axons in the bundle. For each OSN, the ventroposterior point of entry into the dataset was tagged as the root node, and the first branch point of the axonal arbor was tagged “first branch point”. The OSNs’ first branch point was typically close to where the OSN entered and started to branch and ramify in its home glomerulus. We identified the uPN axons in the projection nerve bundle (iACT, S1B Fig) from the antennal lobe to higher brain centers. Tracing these back until they entered glomeruli, we found one densely branching uPN innervated each of the three maxillary palp glomeruli. PNs projecting through other tracts were not included in this analysis. Each uPN had a large-caliber process leading laterally to a cortical soma cluster, though only Glomerulus 1 uPN’s soma was included in the bounds of the dataset. A multiglomerular PN (mPN) was identified by an axon in the iACT with a process projecting laterally towards a soma, and innervation of two different antennal lobe glomeruli.

In OSNs and PNs, T-bar synapses were identified and annotated using ultrastructural criteria: a presynaptic T-bar, presynaptic vesicles, synaptic cleft, and postsynaptic densities [45,47]. We identified *en face* cut synapses when a section contained a presynaptic specialization and an adjacent section(s) contained vesicle pools, and putative postsynaptic cells contained postsynaptic densities. Putative ribbon synapses consisted of a presynaptic density oriented non-parallel to the presynaptic membrane that was surrounded on all sides by vesicles (S10 and S11 Figs and S2 Table). The vast majority occurred at lower branch orders (Figs 5, S10, and S11). Ribbon synapses were differentiated from obliquely cut synapses by vesicle pools present on the same sections as the presynaptic density and the lack of putative postsynaptic cells on sections adjacent to the presynaptic density.

To increase our annotation accuracy, each fully reconstructed OSN and uPN was proofread by a minimum of three trained annotators, who marked all synapses with a connector node and all postsynaptic partners with a new skeleton. We reconstructed all postsynaptic partners of the 30 fully reconstructed OSNs, 10 randomly selected for each glomerulus, to identify their cell type. To empirically validate that the selected OSNs morphologies were representative, we used NBLAST to generate similarity scores for all OSNs in each glomerulus [56]. Neurons not identified as OSNs or uPNs are referred to here as MGs. Operationally, MGs were reconstructed cells that extended beyond their home glomerulus. Most of these are likely LNs, because this cell type is the most numerous and broadly arborizing of the MGs in the insect antennal lobe [108,109]. Additionally, some of these may be mPNs, or neurons that project into the antennal lobe from other regions. Some presynaptic specializations (<10%) appeared to contact glia or to interstitial space between cells.

### Connectivity analysis

Analysis was performed in Python using pymaid (version 2.4.0, https://pymaid.readthedocs.io) and NAVis (version 1.5.0, https://navis.readthedocs.io). Data visualization and plotting were performed using matplotlib (version 3.8.3) and seaborn (version 0.13.2) packages and statistics were performed using SciPy (version 1.12.0), with posthoc tests performed using scikit-posthocs (version 0.9.0).

To measure cable length and distances between synapses and branch points, we computed geodesic or “along the arbor” distances. Because the opportunities for neurons to be synaptically connected is limited to regions where neuron processes are close enough to make synapses, we control for this by computing synapse density. We define synapse density as the number of presynaptic output synapses normalized by the cable overlap, which is the length of presynaptic neurite within sufficiently close proximity (<2 µm) of the postsynaptic neurite to make a synapse.

To control for differences in neurite lengths from each glomerulus to where axons reach the boundaries of the EM volume, we used two approaches to restrict our analysis to within glomeruli themselves. First, we generated meshes that defined the 3D boundary of the glomeruli and limited our analyses to neurites and synapses within the bounds of the 3D meshes. Second, for OSNs, we restricted our analysis to branches and synapses distal from the first branch point of the OSN axon, which was typically near where the axon enters the glomerulus.

### Axogram and dendrograms

Axograms and dendrograms were generated with Neuroboom [36] (version 0.3.55, https://github.com/markuspleijzier/neuroboom). OSNs were plotted vertically (dot progression), whereas PNs were plotted radially (neato progression) to embed their more complex arbors.

### Additional technical notes on reconstructed neurons

We identified a single putative mPN that sparsely innervates Glomerulus 1 and another unidentified glomerulus. This neuron has an axon tract that exits the antennal lobe in iACT, and may be an mPN. The branches were reconstructed, but it was not used in inter-glomerular comparative analyses because we were unable to do a complete survey of mPNs without a larger EM volume. We do not rule out the existence of other PNs that may exit through separate tracts [33,110,111].

### Analysis of *D. melanogaster* neurons in Flywire

To compare *Ae aegypti* glomerular wiring and connectivity to that of *D. melanogaster*, we first identified antennal lobe glomeruli in the Flywire dataset [28,67]. We then proofread OSNs of the CO_2_-selective glomerulus V and appetitive glomerulus DM1 on the right side of the brain for merge and split errors; we selected the right side was because at the time it contained better-proofread neurons. Subsequent proofreading by the FlyWire consortium completed the rest of the OSNs and PNs [28,29]. We analyzed fly OSNs, PNs, and their connectivity in the right hemisphere using the glomerular boundary meshes made previously [32]. We used fafbseg (version 3.0.10; https://fafbseg-py.readthedocs.io) to pull data from the 3D reconstructions and the skeletonization function to generate skeletons from 3D neuron meshes. Specifically, OSNs were identified using the hemibrain_type field for all glomeruli except VC3, VC5, and VM6 which utilized the cell_type field due to changes in FlyWire’s data structure. Additionally, we removed cells (*N* = 33) labeled as OSNs from the analysis if they did not have any synapses onto a PN. For the purposes of comparison, we used a previously compiled list of uPNs [33]. We then used the flywire.skid_to_id function to recover the root_id in the FlyWire dataset.

In Flywire, a synapse prediction network [112] assigns each predicted synapse a cleft score, higher cleft scores reduce the chance of a false positive result, though the likelihood of a false negative result also increases. To estimate a cleft score for Flywire antennal lobe synapses with optimal and comparable accuracy, we manually inspected a sample of 19 feedforward OSN to PN synapses in V. We found a cleft score = 65 that provided the highest *f*-score = 0.91, with precision = 0.88 and recall = 0.94. This means ~6% of manually annotated synapses are missed, with slightly more false positives. We used a cleft score of 65 for the purposes of this analysis. To remove instances of nonexistent autapses, we set the parameter filter = True regardless of cleft score.

### Quantification and statistical analysis

Unless otherwise stated, we used two-tailed Kruskal–Wallis tests and Dunn’s posthoc tests with Bonferroni correction for pairwise comparisons. We implemented an algorithm (Piepho, 2004, [113]) that utilizes pairwise comparisons to identify significance groupings (S7 and S8 Figs). We used the non-parametric Kruskal–Wallis test to statistically compare across more than two groups because we do not assume normality and it is more robust to outliers. Some *D. melanogaster* glomeruli exhibit extreme values (Figs 3, 4, S7, and S8). Since normality was not assumed, a formal statistical test for outliers was not applicable. However, we inspected all data for points where synapse density was greater than two median absolute deviations (2 × MADs) from the median. All feedforward synapses that were 2 × MAD from the median that we visually inspected were verified as synapses, and these data were included in all analyses. We identified only 4 false-positive recurrent synapses among the outlier population. To most accurately reflect the morphology, these synapses were removed from the data presented throughout the paper. This difference did not change any of the statistically significant findings. The locations of the synapses in the FlyWire dataset that were removed are:

**Table:** 

glomerulus	presynaptic cell_ID	presynaptic cell_ID	x location	y location	z location
VL2a	720575940627452668	720575940629918459	105268	59425	1582
DL3	720575940630014917	720575940621914392	110655	51685	1236
VM3	720575940638434357	720575940628897280	136581	64867	846
VL1	720575940629587031	720575940631025103	107970	65322	1650

### Network model

We simulated our model in Python using custom software. A notebook for simulating the modeling results are available on GitHub or Zenodo: https://github.com/htem/aedes_public (or https://doi.org/10.5281/zenodo.21403889). Model parameters were selected based on previous work [71–73]. We used values of *σ* = 12 spikes/s and *R*_*max*_ = 165 spikes/s based on OSN and PN models fit to experimental data [71]. The exponent 1.5 was used to simulate CO_2_ in the presence of other odors (‘background odors’) that broadly activated many glomeruli [71]. We set *s* = 50 spikes/s. This value was consistent with models [71,72], and also with a weighted average OSN response of 43 sp/s calculated from 186 odor responses from 24 *D. melanogaster* glomeruli [73]. To select PN and OSN threshold values for our detection task, we used previous experimental findings. To estimate the PN threshold, we computed the average PN response across a distribution of odors [73] and selected this threshold to be two standard deviations larger than this mean. We assumed PN responses around this mean were Gaussian distributed with previously derived variance parameters [72]. To compute the OSN threshold of 35 sp/s, we considered experiments with multiple mosquito species including *Ae. aegypti* that found that in an environment devoid of background CO_2_, OSNs were unresponsive to CO_2_ concentrations less than 200 ppm CO_2_ but responded at a rate of approximately 35 sp/s for concentrations of 300 ppm [114,115], which they noted corresponds to the ambient CO_2_ level in natural environments. Additionally, experimental work also found OSN response rates of around 35 sp/s for CO_2_ concentrations 200 ppm greater than background CO_2_ concentrations, for a variety of background levels [74]. Values of *g* identified in Fig 6E were found using scipy’s root-finding method, root_scalar.

### Gene expression of Acetylcholine and GABA in olfactory sensory neurons

We analyzed levels of gene expression in OSNs from neurons in both the maxillary palps and antennae by retrieving snRNA-seq data found in the Mosquito Cell Atlas [70]. Cells were categorized by their previously annotated subtypes (CpA cells, CpB cells, CpC cell) along with the tissue that they were isolated from (maxillary palps or antennae). To identify whether a given gene was expressed or not by a particular subtype, we used the average raw expression level per cell, where anything above 10 counts per cell is labelled as 10+ and anything less than one is labelled as 0. We restricted our analysis to the orthologues of the *Drosophila melanogaster* Acetylcholine and GABA receptors along with their associated processing enzymes. The information for each gene is provided in S1 Table.

## Supporting information

S1 Fig MicroCT and PNs with axons in iACT. **(A)** X-ray micro-computed tomography (microCT) of the *Ae. aegypti* brain analyzed in this study (anterior view). Scale bar 100 µm. **(B)** Coronal *(left)* and sagittal *(right)* views of all PNs on the left side of the brain that contained axons that fasciculate into the ipsilateral iACT. Ispilateral iACT uPNs were traced to completion while putative multiglomerular cells were not. a, anterior; d, dorsal; l, lateral; m, medial; p, posterior; v, ventral. Scale bar 50 µm.
(EPS)

S2 Fig Examples of T-bar synapses and postsynaptic partners in *Aedes aegypti.* **(A)** Zoomed out (top) and zoomed in (bottom) canonical T-bar synapses, indicated by blue and red arrows respectively. Here, both synapses are characterized by the clustering of vesicles, postsynaptic densities, and presynaptic T-bar structures. **(B)** Example of one presynaptic cell making two closely located synapses. These are considered separately as one (blue arrow) synapse’s characteristics disappear for at least 40 nm before appearing for the subsequent nearby synapse (red arrow). **(C)** Example of one T-bar synapse (blue arrow) and its postsynaptic partners. Presynaptic cell in yellow, postsynaptic partners in blue. Partners not considered postsynaptic for this synapse - based on contact with the synaptic cleft—are marked in red. Scale bars 500 nm.
(EPS)

S3 Fig Reconstructed OSN morphologies. Skeletonized neuron morphologies are shown for the 10 OSNs fully reconstructed from Glomerulus 1, 2, and 3. Labels are neuron IDs corresponding to the CATMAID database. Neuron 7,136 had a proximal branch that projects dorsoposteriorly toward higher brain areas. Colored dots indicate synapse type (key bottom-right). a, anterior; d, dorsal; l, lateral; m, medial; p, posterior; v, ventral. Scale bar 25 µm.
(EPS)

S4 Fig Dorso-posteriorly projecting tract of OSN processes co-fasciculates with the iACT. **(A)** Rendering of 5 OSNs (colored traces) innervating Glomerulus 1 and Glomerulus 2 (gray volumes represent glomerular boundaries; Glomerulus 3 is also depicted, but none of the extra-projectional OSNs innervate it) that send small-caliber neurites along the iACT (medial uPN tract, Supplementary Fig 1B) to higher-order brain regions. (top) Right side of brain. (bottom) Left side of brain. Dashed lines indicate locations of EM micrographs shown in (B). a, anterior; d, dorsal; l, lateral; m, medial; p, posterior; v, ventral. Scale bar 25 µm. **(B)** EM micrograph at dashed line locations in (A) Co-fasciculating neurites indicated by arrows. Scale bars 1 µm.
(EPS)

S5 Fig Analyses of connectivity including extraglomerular OSN and uPN axons. **(A)** OSN cable length within each glomerulus. (Kruskal–Wallis test (two-tailed), *p* = 1.81 × 10^−5^, pairwise comparisons using Dunn’s post-hoc test with Bonferroni correction: n.s., *p* > 0.05; * *p* ≤ 0.01; ** *p* ≤ 0.001). **(B)** Number of branches for OSNs in each glomerulus. (Kruskal–Wallis test (two-tailed), *p* = 6.32 × 10^−5^, pairwise comparisons using Dunn’s post-hoc test with Bonferroni correction: n.s., *p* > 0.05; * *p* ≤ 0.01; ** *p* ≤ 0.001). **(C)** Mean number of branches at each branch order for OSNs in each glomerulus. **(D)** Number of OSN-to-uPN synapses (Kruskal–Wallis test (two-tailed), *p* = 0.0002, pairwise comparisons using Dunn’s posthoc test with Bonferroni correction: n.s., *p* > 0.05; * *p* ≤ 0.01). **(E)** Density of OSN-to-uPN synapses (Kruskal–Wallis test (two-tailed), *p* = 3.61 × 10^−5^, pairwise comparisons using Dunn’s post-hoc test with Bonferroni correction: n.s., *p* > 0.05; * *p* ≤ 0.05; ** *p* ≤ 0.001). **(F)** Total number of outgoing OSN-to-OSN synapses restricted to within glomerular boundaries (Kruskal–Wallis test (two-tailed), *p* = 1.28 × 10^−5^, pairwise comparisons using Dunn’s post-hoc test with Bonferroni correction: n.s., *p* > 0.05; * *p* ≤ 0.05; ** *p* ≤ 0.001). **(G)** Density of OSN-to-OSN synapses to within glomerular boundaries (Kruskal–Wallis test (two-tailed), *p* = 3.65 × 10^−6^, pairwise comparisons using Dunn’s post-hoc test with Bonferroni correction: n.s., *p* > 0.05; * *p* ≤ 0.01). The data underlying this Figure can be found in S1–S3, S5, S6, S9, and S10 Data.
(EPS)

S6 Fig Visualization of statistical significance and median difference of morphological metrics of *D. melanogaster* and Glomerulus 1. Representation of each glomerulus is composed of two parts: size and color. The size of each point is the linearly scaled absolute difference of each metric. The color of each point is the significance of the pairwise statistical test for each glomerulus. **(A)** Color bar where significance from 0 to 0.05 is scaled at a different linear rate than from 0.05 to 1 to emphasize different degrees of significance. Red hues represent significance and blue hues represent non-significance. **(B)** Median difference of cable length within each glomerulus from Glomerulus 1, significance as indicated in (A). **(C)** Median difference of feedforward metrics of each glomerulus from Glomerulus 1, significance as indicated in (A). **(D)** Median difference of recurrent metrics of each glomerulus from Glomerulus 1, significance as indicated in (A). The data underlying this Figure can be found in S1–S7 Data.
(EPS)

S7 Fig Pairwise analysis of feedforward connectivity for all glomeruli. **(A)** Number of OSN-to-uPN synapses restricted to within glomerular boundaries (Kruskal–Wallis test (two-tailed), *p* = 9.21 × 10^−140^, different letters mark whether glomeruli are significantly different using Dunn’s posthoc test with Bonferroni correction: *p* ≤ 0.01). **(B)** Density of OSN-to-uPN synapses within glomerular boundaries (Kruskal–Wallis test (two-tailed), *p* = 1.32 × 10^−137^, compact letter display as in (A)). **(C)** OSN-to-uPN synapses restricted to within glomerular boundaries as a fraction of overall OSN output synapses (Kruskal–Wallis test (two-tailed), *p* = 1.63 × 10^−91^, compact letter display as in (a)). The data underlying this Figure can be found in S5–S7 Data.
(EPS)

S8 Fig Analyses of recurrent connectivity for all glomeruli. **(A)** Number of OSN-to-OSN synapses restricted to within glomerular boundaries (Kruskal–Wallis test (two-tailed), *p* = 7.88 × 10^−105^, different letters mark whether glomeruli are significantly different using Dunn’s posthoc test with Bonferroni correction: *p* ≤ 0.01). **(B)** Density of OSN-to-OSN synapses within glomerular boundaries (Kruskal–Wallis test (two-tailed), *p* = 6.96 × 10^−78^, compact letter display as in (A)). **(C)** OSN-to-OSN synapses restricted to within glomerular boundaries as a fraction of overall OSN output synapses (Kruskal–Wallis test (two-tailed), *p* = 1.03 × 10^−79^, compact letter display as in (a)). The data underlying this Figure can be found in S2–S4 Data.
(EPS)

S9 Fig Reconstructions of OSNs and uPNs of *D. melanogaster* glomerulus V. **(A)** V OSNs and uPNs reconstructed in Flywire [28,29,33]. Lighter shades of orange and purple correspond to OSNs, while darker shades correspond to uPNs. Scale bars 50 µm. **(B)** Number of OSN to uPN synapses restricted to within glomerular boundaries (Kruskal–Wallis test (two-tailed), *p* = 5.17 × 10^−17^, different letters mark whether glomeruli are significantly different using Dunn’s posthoc test with Bonferroni correction: *p* ≤ 0.01). **(C)** Density of OSN to uPN synapses within glomerular boundaries (Kruskal–Wallis test (two-tailed), *p* = 1.62 × 10^−09^, letters display significant differences as in (B)). **(D)** snRNAseq data replotted from [70], for acetylcholine processing enzymes and (E) receptors. The data underlying this Figure can be found in S5 and S6 Data.
(EPS)

S10 Fig Examples of ribbon synapses in vertebrates. **(A–C)** Schematics of ribbon synapses across vertebrate species. (A) Ribbon synapse in rabbit retina between rod bipolar cells and amacrine cells [53]. (B) Ribbon synapse in a zebrafish lateral line hair cell [52]. (C) Ribbon synapse in mouse auditory hair cell [51].
(EPS)

S11 Fig Serial-sections of ribbon-like structures. Serial electron micrographs through ribbon-like structures in Glomerulus 1 OSNs. Labels are connector IDs corresponding to the CATMAID database. Scale bars 1 µm. See S2 Table for links to view image data for each putative ribbon synapse in CATMAID. Scale bars 1 µm.
(EPS)

S1 Table This table includes the full gene names for the genes that are analyzed in the snRNAseq plots, gene abbreviations, corresponding AAEL numbers for identification in the *Ae. aegypti* genome, and FlyBase Gene Accession Numbers for the nearest gene orthologues in *D. melanogaster.* (XLSX)

S2 Table Links to view aligned EM image data with putative ribbon synapses in S11 Fig. (XLSX)

S1 Data Data in Figs 1H, 3E, S5A, and S6B. (CSV)

S2 Data Data in Figs 1L, 4B, S5F, S6D, and S8A. (CSV)

S3 Data Data in Figs 1M, 4C, S5G, S6D, and S8B. (CSV)

S4 Data Data in Figs 1N, 4D, S6D, and S8C. (CSV)

S5 Data Data in Figs 2E, 3B, S5D, S6C, S7A, and S9B. (CSV)

S6 Data Data in Figs 2F, 3C, S5E, S6C, S7B, and S9C. (CSV)

S7 Data Data in Figs 2G, 3D, S6C, and S7C. (CSV)

S8 Data Data in Fig 2H. (CSV)

S9 Data Data in Figs 5G and S5C. (CSV)

S10 Data Data in Figs 5H and S5B. (CSV)

## Abbreviations

ALs: antennal lobes
ATUM: automated tape-collecting microtome
EM: electron microscopy
GR: gustatory receptor
iACT: inner antennocerebral tract
LNs: local neurons
MD: medio-dorsal
MGs: multiglomerular neurons
mPN: multiglomerular PN
OSNs: olfactory sensory neurons
PBS: phosphate-buffered saline
PNs: projection neurons
ROIs: regions of interest
uPNs: uniglomerular PNs

## References

[1] Vector-borne diseases. World Health Organization. 2023.

[2] Iwamura T, Guzman-Holst A, Murray KA. Accelerating invasion potential of disease vector *Aedes aegypti* under climate change. Nat Commun. 2020;11(1):2130. doi: 10.1038/s41467-020-16010-4 32358588 PMC7195482

[3] Lee J-S, Farlow A. The threat of climate change to non-dengue-endemic countries: increasing risk of dengue transmission potential using climate and non-climate datasets. BMC Public Health. 2019;19(1):934. doi: 10.1186/s12889-019-7282-3 31296193 PMC6625070

[4] Christophers SR. *Aedes aegypti*, the yellow fever mosquito; its life history, bionomics, and structure. Cambridge University Press; 1960.

[5] Zhao Z, Zung JL, Hinze A, Kriete AL, Iqbal A, Younger MA, et al. Mosquito brains encode unique features of human odour to drive host seeking. Nature. 2022;605(7911):706–12. doi: 10.1038/s41586-022-04675-4 35508661 PMC9725754

[6] Dekker T, Geier M, Cardé RT. Carbon dioxide instantly sensitizes female yellow fever mosquitoes to human skin odours. J Exp Biol. 2005;208(Pt 15):2963–72. doi: 10.1242/jeb.01736 16043601

[7] McMeniman CJ, Corfas RA, Matthews BJ, Ritchie SA, Vosshall LB. Multimodal integration of carbon dioxide and other sensory cues drives mosquito attraction to humans. Cell. 2014;156(5):1060–71. doi: 10.1016/j.cell.2013.12.044 24581501 PMC4007582

[8] Liu MZ, Vosshall LB. General visual and contingent thermal cues interact to elicit attraction in female *Aedes aegypti* mosquitoes. Curr Biol. 2019;29(13):2250-2257.e4. doi: 10.1016/j.cub.2019.06.001 31257144

[9] Corfas RA, Vosshall LB. The cation channel TRPA1 tunes mosquito thermotaxis to host temperatures. Elife. 2015;4:e11750. doi: 10.7554/eLife.11750 26670734 PMC4718722

[10] Laursen WJ, Budelli G, Tang R, Chang EC, Busby R, Shankar S, et al. Humidity sensors that alert mosquitoes to nearby hosts and egg-laying sites. Neuron. 2023;111(6):874-887.e8. doi: 10.1016/j.neuron.2022.12.025 36640768 PMC10023463

[11] Khan AA, Maibach HI. Quantitation of effect of several stimuli on landing and probing by *Aedes aegypti*. J Econ Entomol. 1966;59:902–5. doi: 10.1093/jee/59.4.902 5932265

[12] Vinauger C, Van Breugel F, Locke LT, Tobin KKS, Dickinson MH, Fairhall AL, et al. Visual-olfactory integration in the human disease vector mosquito *Aedes aegypti*. Curr Biol. 2019;29(15):2509-2516.e5. doi: 10.1016/j.cub.2019.06.043 31327719 PMC6771019

[13] Xu P, Wen X, Leal WS. CO2 per se activates carbon dioxide receptors. Insect Biochem Mol Biol. 2020;117:103284. doi: 10.1016/j.ibmb.2019.103284 31760135 PMC6980743

[14] Dekker T, Cardé RT. Moment-to-moment flight manoeuvres of the female yellow fever mosquito (*Aedes aegypti* L.) in response to plumes of carbon dioxide and human skin odour. J Exp Biol. 2011;214(Pt 20):3480–94. doi: 10.1242/jeb.055186 21957112

[15] Lacey ES, Ray A, Cardé RT. Close encounters: contributions of carbon dioxide and human skin odour to finding and landing on a host in *Aedes aegypti*. Physiol Entomol. 2014;39(1):60–8. doi: 10.1111/phen.12048 24839345 PMC4019456

[16] Matthews BJ, Younger MA, Vosshall LB. The ion channel ppk301 controls freshwater egg-laying in the mosquito *Aedes aegypti*. Elife. 2019;8:e43963. doi: 10.7554/eLife.43963 31112133 PMC6597239

[17] Heinze S, El Jundi B, Berg BG, Homberg U, Menzel R, Pfeiffer K, et al. A unified platform to manage, share, and archive morphological and functional data in insect neuroscience. Elife. 2021;10:e65376. doi: 10.7554/eLife.65376 34427185 PMC8457822

[18] Phelps JS, Hildebrand DGC, Graham BJ, Kuan AT, Thomas LA, Nguyen TM, et al. Reconstruction of motor control circuits in adult *Drosophila* using automated transmission electron microscopy. Cell. 2021;184(3):759-774.e18. doi: 10.1016/j.cell.2020.12.013 33400916 PMC8312698

[19] Couto A, Alenius M, Dickson BJ. Molecular, anatomical, and functional organization of the *Drosophila* olfactory system. Curr Biol. 2005;15(17):1535–47. doi: 10.1016/j.cub.2005.07.034 16139208

[20] Fishilevich E, Vosshall LB. Genetic and functional subdivision of the *Drosophila* antennal lobe. Curr Biol. 2005;15(17):1548–53. doi: 10.1016/j.cub.2005.07.066 16139209

[21] Stocker RF, Lienhard MC, Borst A, Fischbach KF. Neuronal architecture of the antennal lobe in *Drosophila melanogaster*. Cell Tissue Res. 1990;262(1):9–34. doi: 10.1007/BF00327741 2124174

[22] Stocker RF, Singh RN, Schorderet M, Siddiqi O. Projection patterns of different types of antennal sensilla in the antennal glomeruli of *Drosophila melanogaster*. Cell Tissue Res. 1983;232(2):237–48. doi: 10.1007/BF00213783 6411344

[23] Wilson RI. Early olfactory processing in Drosophila: mechanisms and principles. Annu Rev Neurosci. 2013;36:217–41. doi: 10.1146/annurev-neuro-062111-150533 23841839 PMC3933953

[24] Tobin WF, Wilson RI, Lee W-CA. Wiring variations that enable and constrain neural computation in a sensory microcircuit. Elife. 2017;6:e24838. doi: 10.7554/eLife.24838 28530904 PMC5440167

[25] Horne JA, Langille C, McLin S, Wiederman M, Lu Z, Xu CS, et al. A resource for the *Drosophila* antennal lobe provided by the connectome of glomerulus VA1v. Elife. 2018;7:e37550. doi: 10.7554/eLife.37550 30382940 PMC6234030

[26] Gruber L, Cantera R, Pleijzier MW, Steinert M, Pertsch T, Hansson BS. The unique synaptic circuitry of specialized olfactory glomeruli in *Drosophila melanogaster*. 2023. doi: 10.7554/eLife.88824.1 PMC12537010 41114722

[27] Scheffer LK, Xu CS, Januszewski M, Lu Z, Takemura S-Y, Hayworth KJ, et al. A connectome and analysis of the adult *Drosophila* central brain. Elife. 2020;9:e57443. doi: 10.7554/eLife.57443 32880371 PMC7546738

[28] Dorkenwald S, Matsliah A, Sterling AR, Schlegel P, Yu S-C, McKellar CE, et al. Neuronal wiring diagram of an adult brain. Nature. 2024;634(8032):124–38. doi: 10.1038/s41586-024-07558-y 39358518 PMC11446842

[29] Schlegel P, Yin Y, Bates AS, Dorkenwald S, Eichler K, Brooks P, et al. Whole-brain annotation and multi-connectome cell typing of *Drosophila*. Nature. 2024;634(8032):139–52. doi: 10.1038/s41586-024-07686-5 39358521 PMC11446831

[30] Zheng Z, Lauritzen JS, Perlman E, Robinson CG, Nichols M, Milkie D, et al. A complete electron microscopy volume of the brain of adult *Drosophila melanogaster*. Cell. 2018;174(3):730-743.e22. doi: 10.1016/j.cell.2018.06.019 30033368 PMC6063995

[31] Frechter S, Bates AS, Tootoonian S, Dolan M-J, Manton J, Jamasb AR, et al. Functional and anatomical specificity in a higher olfactory centre. Elife. 2019;8:e44590. doi: 10.7554/eLife.44590 31112127 PMC6550879

[32] Schlegel P, Bates AS, Stürner T, Jagannathan SR, Drummond N, Hsu J, et al. Information flow, cell types and stereotypy in a full olfactory connectome. Elife. 2021;10:e66018. doi: 10.7554/eLife.66018 34032214 PMC8298098

[33] Bates AS, Schlegel P, Roberts RJV, Drummond N, Tamimi IFM, Turnbull R, et al. Complete connectomic reconstruction of olfactory projection neurons in the fly brain. Curr Biol. 2020;30(16):3183-3199.e6. doi: 10.1016/j.cub.2020.06.042 32619485 PMC7443706

[34] Dolan M-J, Frechter S, Bates AS, Dan C, Huoviala P, Roberts RJ, et al. Neurogenetic dissection of the *Drosophila* lateral horn reveals major outputs, diverse behavioural functions, and interactions with the mushroom body. Elife. 2019;8:e43079. doi: 10.7554/eLife.43079 31112130 PMC6529221

[35] Huoviala P, Dolan MJ, Love FM, Myers P, Frechter S, Namiki S. Neural circuit basis of aversive odour processing in Drosophila from sensory input to descending output. bioRxiv. 2020. doi: 10.1101/394403

[36] Felsenberg J, Jacob PF, Walker T, Barnstedt O, Edmondson-Stait AJ, Pleijzier MW, et al. Integration of parallel opposing memories underlies memory extinction. Cell. 2018;175(3):709-722.e15. doi: 10.1016/j.cell.2018.08.021 30245010 PMC6198041

[37] da Silva AF, Machado LC, de Paula MB, da Silva Pessoa Vieira CJ, de Morais Bronzoni RV, de Melo Santos MAV, et al. Culicidae evolutionary history focusing on the Culicinae subfamily based on mitochondrial phylogenomics. Sci Rep. 2020;10(1):18823. doi: 10.1038/s41598-020-74883-3 33139764 PMC7606482

[38] Arensburger P, Megy K, Waterhouse RM, Abrudan J, Amedeo P, Antelo B, et al. Sequencing of *Culex quinquefasciatus* establishes a platform for mosquito comparative genomics. Science. 2010;330(6000):86–8. doi: 10.1126/science.1191864 20929810 PMC3740384

[39] Herre M, Goldman OV, Lu T-C, Caballero-Vidal G, Qi Y, Gilbert ZN, et al. Non-canonical odor coding in the mosquito. Cell. 2022;185(17):3104-3123.e28. doi: 10.1016/j.cell.2022.07.024 35985288 PMC9480278

[40] Fernández-Chiappe F, Ocker GK, Younger MA. Prospects on non-canonical olfaction in the mosquito and other organisms: why co-express?. Curr Opin Insect Sci. 2025;67:101291. doi: 10.1016/j.cois.2024.101291 39471910

[41] Südhof TC. The synaptic vesicle cycle. Annu Rev Neurosci. 2004;27:509–47. doi: 10.1146/annurev.neuro.26.041002.131412 15217342

[42] Harris KP, Littleton JT. Transmission, development, and plasticity of synapses. Genetics. 2015;201:345–75. doi: 10.1534/genetics.115.176529 26447126 PMC4596655

[43] Südhof TC. The presynaptic active zone. Neuron. 2012;75(1):11–25. doi: 10.1016/j.neuron.2012.06.012 22794257 PMC3743085

[44] Matthews G, Fuchs P. The diverse roles of ribbon synapses in sensory neurotransmission. Nat Rev Neurosci. 2010;11(12):812–22. doi: 10.1038/nrn2924 21045860 PMC3065184

[45] Prokop A, Meinertzhagen IA. Development and structure of synaptic contacts in *Drosophila*. Semin Cell Dev Biol. 2006;17(1):20–30. doi: 10.1016/j.semcdb.2005.11.010 16384719

[46] Sterling P, Matthews G. Structure and function of ribbon synapses. Trends Neurosci. 2005;28(1):20–9. doi: 10.1016/j.tins.2004.11.009 15626493

[47] Wagh DA, Rasse TM, Asan E, Hofbauer A, Schwenkert I, Dürrbeck H, et al. Bruchpilot, a protein with homology to ELKS/CAST, is required for structural integrity and function of synaptic active zones in Drosophila. Neuron. 2006;49:833–44. doi: 10.1016/j.neuron.2006.02.008 16543132

[48] Kantardzhieva A, Peppi M, Lane WS, Sewell WF. Protein composition of immunoprecipitated synaptic ribbons. J Proteome Res. 2012;11(2):1163–74. doi: 10.1021/pr2008972 22103298 PMC3274173

[49] Magupalli VG, Schwarz K, Alpadi K, Natarajan S, Seigel GM, Schmitz F. Multiple RIBEYE-RIBEYE interactions create a dynamic scaffold for the formation of synaptic ribbons. J Neurosci. 2008;28(32):7954–67. doi: 10.1523/JNEUROSCI.1964-08.2008 18685021 PMC6670776

[50] Schmitz F, Königstorfer A, Südhof TC. RIBEYE, a component of synaptic ribbons. Neuron. 2000;28(3):857–72. doi: 10.1016/s0896-6273(00)00159-8 11163272

[51] Nouvian R, Beutner D, Parsons TD, Moser T. Structure and function of the hair cell ribbon synapse. J Membr Biol. 2006;209(2–3):153–65. doi: 10.1007/s00232-005-0854-4 16773499 PMC1764598

[52] Kindt KS, Sheets L. Transmission disrupted: modeling auditory synaptopathy in zebrafish. Front Cell Dev Biol. 2018;6:114. doi: 10.3389/fcell.2018.00114 30258843 PMC6143809

[53] Raviola E, Dacheux RF. Excitatory dyad synapse in rabbit retina. Proc Natl Acad Sci U S A. 1987;84(20):7324–8. doi: 10.1073/pnas.84.20.7324 3478695 PMC299285

[54] Ignell R, Dekker T, Ghaninia M, Hansson BS. Neuronal architecture of the mosquito deutocerebrum. J Comp Neurol. 2005;493(2):207–40. doi: 10.1002/cne.20800 16255032

[55] Mclver SB. Sensilla of mosquitoes (Diptera: Culicidae)1, 2. J Med Entomol. 1982;19(5):489–535. doi: 10.1093/jmedent/19.5.489 6128422

[56] Costa M, Manton JD, Ostrovsky AD, Prohaska S, Jefferis GSXE. NBLAST: rapid, sensitive comparison of neuronal structure and construction of neuron family databases. Neuron. 2016;91(2):293–311. doi: 10.1016/j.neuron.2016.06.012 27373836 PMC4961245

[57] Holler S, Köstinger G, Martin KAC, Schuhknecht GFP, Stratford KJ. Structure and function of a neocortical synapse. Nature. 2021;591(7848):111–6. doi: 10.1038/s41586-020-03134-2 33442056

[58] Nicoll RA. Recurrent excitation of secondary olfactory neurons: a possible mechanism for signal amplification. Science. 1971;171(3973):824–6. doi: 10.1126/science.171.3973.824 5541166

[59] Douglas RJ, Koch C, Mahowald M, Martin KA, Suarez HH. Recurrent excitation in neocortical circuits. Science. 1995;269(5226):981–5. doi: 10.1126/science.7638624 7638624

[60] Faucher C, Forstreuter M, Hilker M, de Bruyne M. Behavioral responses of *Drosophila* to biogenic levels of carbon dioxide depend on life-stage, sex and olfactory context. J Exp Biol. 2006;209(Pt 14):2739–48. doi: 10.1242/jeb.02297 16809465

[61] Suh GSB, Wong AM, Hergarden AC, Wang JW, Simon AF, Benzer S, et al. A single population of olfactory sensory neurons mediates an innate avoidance behaviour in *Drosophila*. Nature. 2004;431(7010):854–9. doi: 10.1038/nature02980 15372051

[62] Wasserman S, Salomon A, Frye MA. Drosophila tracks carbon dioxide in flight. Curr Biol. 2013;23(4):301–6. doi: 10.1016/j.cub.2012.12.038 23352695 PMC3810385

[63] Lin H-H, Chu L-A, Fu T-F, Dickson BJ, Chiang A-S. Parallel neural pathways mediate CO 2 avoidance responses in *Drosophila*. Science. 2013;340(6138):1338–41. doi: 10.1126/science.1236693 23766327

[64] van Breugel F, Huda A, Dickinson MH. Distinct activity-gated pathways mediate attraction and aversion to CO2 in *Drosophila*. Nature. 2018;564(7736):420–4. doi: 10.1038/s41586-018-0732-8 30464346 PMC6314688

[65] Jones WD, Cayirlioglu P, Kadow IG, Vosshall LB. Two chemosensory receptors together mediate carbon dioxide detection in *Drosophila*. Nature. 2007;445(7123):86–90. doi: 10.1038/nature05466 17167414

[66] Kwon JY, Dahanukar A, Weiss LA, Carlson JR. The molecular basis of CO2 reception in *Drosophila*. Proc Natl Acad Sci U S A. 2007;104(9):3574–8. doi: 10.1073/pnas.0700079104 17360684 PMC1805529

[67] Dorkenwald S, McKellar CE, Macrina T, Kemnitz N, Lee K, Lu R, et al. FlyWire: online community for whole-brain connectomics. Nat Methods. 2022;19(1):119–28. doi: 10.1038/s41592-021-01330-0 34949809 PMC8903166

[68] Ai M, Min S, Grosjean Y, Leblanc C, Bell R, Benton R, et al. Acid sensing by the *Drosophila* olfactory system. Nature. 2010;468(7324):691–5. doi: 10.1038/nature09537 21085119 PMC3105465

[69] Ai M, Blais S, Park J-Y, Min S, Neubert TA, Suh GSB. Ionotropic glutamate receptors IR64a and IR8a form a functional odorant receptor complex in vivo in *Drosophila*. J Neurosci. 2013;33(26):10741–9. doi: 10.1523/JNEUROSCI.5419-12.2013 23804096 PMC3693055

[70] Goldman OV, DeFoe AE, Qi Y, Jiao Y, Weng S-C, Wick B, et al. A single-nucleus transcriptomic atlas of the adult *Aedes aegypti* mosquito. Cell. 2025;188(25):7267-7290.e26. doi: 10.1016/j.cell.2025.10.008 41172998 PMC12767863

[71] Olsen SR, Bhandawat V, Wilson RI. Divisive normalization in olfactory population codes. Neuron. 2010;66(2):287–99. doi: 10.1016/j.neuron.2010.04.009 20435004 PMC2866644

[72] Luo SX, Axel R, Abbott LF. Generating sparse and selective third-order responses in the olfactory system of the fly. Proc Natl Acad Sci U S A. 2010;107(23):10713–8. doi: 10.1073/pnas.1005635107 20498080 PMC2890779

[73] Hallem EA, Carlson JR. Coding of odors by a receptor repertoire. Cell. 2006;125(1):143–60. doi: 10.1016/j.cell.2006.01.050 16615896

[74] Majeed S, Hill SR, Ignell R. Impact of elevated CO2 background levels on the host-seeking behaviour of *Aedes aegypti*. J Exp Biol. 2014;217(Pt 4):598–604. doi: 10.1242/jeb.092718 24198270

[75] Charara S, Choy J, Cauwenberghs K, Vijayakumar P, Ng R, Kim K-Y, et al. Morphological specializations of mosquito CO2-sensing olfactory receptor neurons. Proc Natl Acad Sci U S A. 2025;122(43):e2514666122. doi: 10.1073/pnas.2514666122 41129220 PMC12582328

[76] Li Y, Ibrahim LA, Liu B, Zhang LI, Tao HW. Linear transformation of thalamocortical input by intracortical excitation. Nat Neurosci. 2013;16(9):1324–30. doi: 10.1038/nn.3494 23933750 PMC3855439

[77] Harris KD, Mrsic-Flogel TD. Cortical connectivity and sensory coding. Nature. 2013;503(7474):51–8. doi: 10.1038/nature12654 24201278

[78] Lien AD, Scanziani M. Tuned thalamic excitation is amplified by visual cortical circuits. Nat Neurosci. 2013;16(9):1315–23. doi: 10.1038/nn.3488 23933748 PMC3774518

[79] Lee W-CA, Bonin V, Reed M, Graham BJ, Hood G, Glattfelder K, et al. Anatomy and function of an excitatory network in the visual cortex. Nature. 2016;532(7599):370–4. doi: 10.1038/nature17192 27018655 PMC4844839

[80] Cossell L, Iacaruso MF, Muir DR, Houlton R, Sader EN, Ko H, et al. Functional organization of excitatory synaptic strength in primary visual cortex. Nature. 2015;518(7539):399–403. doi: 10.1038/nature14182 25652823 PMC4843963

[81] Peron S, Pancholi R, Voelcker B, Wittenbach JD, Ólafsdóttir HF, Freeman J, et al. Recurrent interactions in local cortical circuits. Nature. 2020;579(7798):256–9. doi: 10.1038/s41586-020-2062-x 32132709 PMC8092186

[82] Oldenburg IA, Hendricks WD, Handy G, Shamardani K, Bounds HA, Doiron B, et al. The logic of recurrent circuits in the primary visual cortex. Nat Neurosci. 2024;27(1):137–47. doi: 10.1038/s41593-023-01510-5 38172437 PMC10774145

[83] Cognigni P, Felsenberg J, Waddell S. Do the right thing: neural network mechanisms of memory formation, expression and update in *Drosophila*. Curr Opin Neurobiol. 2018;49:51–8. doi: 10.1016/j.conb.2017.12.002 29258011 PMC5981003

[84] Wilson RI, Laurent G. Role of GABAergic inhibition in shaping odor-evoked spatiotemporal patterns in the *Drosophila* antennal lobe. J Neurosci. 2005;25(40):9069–79. doi: 10.1523/JNEUROSCI.2070-05.2005 16207866 PMC6725763

[85] Debanne D. Information processing in the axon. Nat Rev Neurosci. 2004;5(4):304–16. doi: 10.1038/nrn1397 15034555

[86] Wang XJ. Synaptic reverberation underlying mnemonic persistent activity. Trends Neurosci. 2001;24(8):455–63. doi: 10.1016/s0166-2236(00)01868-3 11476885

[87] Goldman-Rakic PS. Cellular basis of working memory. Neuron. 1995;14(3):477–85. doi: 10.1016/0896-6273(95)90304-6 7695894

[88] Kim SS, Rouault H, Druckmann S, Jayaraman V. Ring attractor dynamics in the *Drosophila* central brain. Science. 2017;356(6340):849–53. doi: 10.1126/science.aal4835 28473639

[89] Hulse BK, Haberkern H, Franconville R, Turner-Evans D, Takemura S-Y, Wolff T, et al. A connectome of the *Drosophila* central complex reveals network motifs suitable for flexible navigation and context-dependent action selection. Elife. 2021;10:e66039. doi: 10.7554/eLife.66039 34696823 PMC9477501

[90] Sorrells TR, Pandey A, Rosas-Villegas A, Vosshall LB. A persistent behavioral state enables sustained predation of humans by mosquitoes. Elife. 2022;11:e76663. doi: 10.7554/eLife.76663 35550041 PMC9154740

[91] Kellogg FE. Water vapour and carbon dioxide receptors in *Aedes aegypti*. J Insect Physiol. 1970;16(1):99–108. doi: 10.1016/0022-1910(70)90117-4 5417711

[92] Manoim-Wolkovitz JE, Camchy T, Rozenfeld E, Chang H-H, Lerner H, Chou Y-H, et al. Nonlinear high-activity neuronal excitation enhances odor discrimination. Curr Biol. 2025;35(7):1521-1538.e5. doi: 10.1016/j.cub.2025.02.034 40107267 PMC11974548

[93] Raviola G, Raviola E. Light and electron microscopic observations on the inner plexiform layer of the rabbit retina. Am J Anat. 1967;120(3):403–25. doi: 10.1002/aja.1001200303 6037326

[94] Maxeiner S, Luo F, Tan A, Schmitz F, Südhof TC. How to make a synaptic ribbon: RIBEYE deletion abolishes ribbons in retinal synapses and disrupts neurotransmitter release. EMBO J. 2016;35(10):1098–114. doi: 10.15252/embj.201592701 26929012 PMC4868958

[95] tom Dieck S, Altrock WD, Kessels MM, Qualmann B, Regus H, Brauner D, et al. Molecular dissection of the photoreceptor ribbon synapse. The Journal of Cell Biology. 2005;168(5):825–36. doi: 10.1083/jcb.200408157 15728193 PMC2171818

[96] Rajashekhar KP, Singh RN. Neuroarchitecture of the tritocerebrum of *Drosophila melanogaster*. J Comp Neurol. 1994;349(4):633–45. doi: 10.1002/cne.903490410 7860793

[97] de Belle JS, Heisenberg M. Associative odor learning in *Drosophila* abolished by chemical ablation of mushroom bodies. Science. 1994;263(5147):692–5. doi: 10.1126/science.8303280 8303280

[98] Connolly JB, Roberts IJ, Armstrong JD, Kaiser K, Forte M, Tully T, et al. Associative learning disrupted by impaired Gs signaling in *Drosophila* mushroom bodies. Science. 1996;274(5295):2104–7. doi: 10.1126/science.274.5295.2104 8953046

[99] Masse NY, Turner GC, Jefferis GS. Olfactory information processing in *Drosophila*. Curr Biol. 2009;19:R700-13. doi: 10.1016/j.cub.2009.06.026 19706282

[100] Matthews BJ, McBride CS, DeGennaro M, Despo O, Vosshall LB. The neurotranscriptome of the *Aedes aegypti* mosquito. BMC Genomics. 2016;17:32. doi: 10.1186/s12864-015-2239-0 26738925 PMC4704297

[101] van Emburg PR, de Bruijn WC. Enhanced cellular membrane contrast in a marine alga by Osmium-azole complexes. Protoplasma. 1984;119(1–2):48–54. doi: 10.1007/bf01287816

[102] Hayworth KJ, Morgan JL, Schalek R, Berger DR, Hildebrand DGC, Lichtman JW. Imaging ATUM ultrathin section libraries with WaferMapper: a multi-scale approach to EM reconstruction of neural circuits. Front Neural Circuits. 2014;8:68. doi: 10.3389/fncir.2014.00068 25018701 PMC4073626

[103] Bock DD, Lee W-CA, Kerlin AM, Andermann ML, Hood G, Wetzel AW, et al. Network anatomy and in vivo physiology of visual cortical neurons. Nature. 2011;471(7337):177–82. doi: 10.1038/nature09802 21390124 PMC3095821

[104] Nguyen TM, Thomas LA, Rhoades JL, Ricchi I, Yuan XC, Sheridan A, et al. Structured cerebellar connectivity supports resilient pattern separation. Nature. 2023;613(7944):543–9. doi: 10.1038/s41586-022-05471-w 36418404 PMC10324966

[105] Shankar S, McMeniman CJ. An updated antennal lobe atlas for the yellow fever mosquito *Aedes aegypti*. PLoS Negl Trop Dis. 2020;14(10):e0008729. doi: 10.1371/journal.pntd.0008729 33079925 PMC7575095

[106] Ito K, Shinomiya K, Ito M, Armstrong JD, Boyan G, Hartenstein V, et al. A systematic nomenclature for the insect brain. Neuron. 2014;81(4):755–65. doi: 10.1016/j.neuron.2013.12.017 24559671

[107] Saalfeld S, Cardona A, Hartenstein V, Tomancak P. CATMAID: collaborative annotation toolkit for massive amounts of image data. Bioinformatics. 2009;25(15):1984–6. doi: 10.1093/bioinformatics/btp266 19376822 PMC2712332

[108] Chou Y-H, Spletter ML, Yaksi E, Leong JCS, Wilson RI, Luo L. Diversity and wiring variability of olfactory local interneurons in the *Drosophila* antennal lobe. Nat Neurosci. 2010;13(4):439–49. doi: 10.1038/nn.2489 20139975 PMC2847188

[109] Lin S, Kao C-F, Yu H-H, Huang Y, Lee T. Lineage analysis of *Drosophila* lateral antennal lobe neurons reveals notch-dependent binary temporal fate decisions. PLoS Biol. 2012;10(11):e1001425. doi: 10.1371/journal.pbio.1001425 23185131 PMC3502534

[110] Tanaka NK, Suzuki E, Dye L, Ejima A, Stopfer M. Dye fills reveal additional olfactory tracts in the protocerebrum of wild-type *Drosophila*. J Comp Neurol. 2012;520(18):4131–40. doi: 10.1002/cne.23149 22592823 PMC4132881

[111] Liang L, Li Y, Potter CJ, Yizhar O, Deisseroth K, Tsien RW, et al. GABAergic projection neurons route selective olfactory inputs to specific higher-order neurons. Neuron. 2013;79(5):917–31. doi: 10.1016/j.neuron.2013.06.014 24012005 PMC3838762

[112] Buhmann J, Sheridan A, Malin-Mayor C, Schlegel P, Gerhard S, Kazimiers T, et al. Automatic detection of synaptic partners in a whole-brain *Drosophila* electron microscopy data set. Nat Methods. 2021;18(7):771–4. doi: 10.1038/s41592-021-01183-7 34168373 PMC7611460

[113] Piepho H-P. An Algorithm for a Letter-Based Representation of All-Pairwise Comparisons. Journal of Computational and Graphical Statistics. 2004;13(2):456–66. 10.1198/1061860043515

[114] Grant AJ, Wigton BE, Aghajanian JG, O’Connell RJ. Electrophysiological responses of receptor neurons in mosquito maxillary palp sensilla to carbon dioxide. J Comp Physiol A. 1995;177(4):389–96. doi: 10.1007/BF00187475 7674195

[115] Syed Z, Leal WS. Maxillary palps are broad spectrum odorant detectors in *Culex quinquefasciatus*. Chem Senses. 2007;32(8):727–38. doi: 10.1093/chemse/bjm040 17569743

