# Supplementary material for: Recurrent synapses between CO2-sensitive olfactory sensory neurons enable robust CO2 detection in Aedes aegypti mosquitoes
Source: PLoS Biol. 2026 Sep 10;24(9):e3003959. doi: 10.1371/journal.pbio.3003959 (PMC13561330; doi:10.1371/journal.pbio.3003959)
Supplement: Supplementary file 1 [file pbio.3003959.s026.pdf]

Response to Reviewers
We thank the referees for their constructive feedback on our manuscript, “Connectivity enabling carbon dioxide sensitivity in the *Aedes aegypti* mosquito." We appreciate the time and effort that went into evaluating our work. We appreciate the reviewers' positive review of the manuscript and recognition of the importance of the study and the resource for the field.

Reviewers found the work an “*...in-depth, high-quality connectomic study executed with clear technical excellence. The work reveals a distinctive circuit organization that likely underlies the salience of CO2…*” Further, the reviewers found the specific work impactful: “*this potentially unique cell biology is particularly striking. The authors' work on these points is convincing, the data is high quality and highly original, and this first foray into using EM connectomic reconstructions to interrogate circuit mechanisms in Aedes will have a strong influence on the field.*” Moreover, the work provides a blueprint and foundation for the field: “*the comparative framework … is particularly appealing and provides important insight at single-synapse resolution.*” and “*the dataset itself is impressive and will be a valuable resource for the field.*”

Referees also raised concerns, which we summarize below, followed by a point-by-point response to the referees' comments. We have revised the manuscript, providing additional analyses and data to address concerns raised by the referees.

1. Reviewers noted that the interpretability of our modeling hinges on the presence of divisive normalization mediated by GABA in Glomerulus 1. To address this add two new analyses: First, we provide new analysis of single cell transcriptomic data with direct evidence for GABA receptor expression in this system (included in Figure 6). Second, we provide new sensitivity analysis with modeling (in the reviewer response) to assess how the conclusions depend on the level of GABA-mediated inhibition. We find that if Glomerulus 1 has weak GABA-B-mediated normalization, recurrent connections are not harmful - and if any normalization is present, they actively ensure robust CO_2 detection across a wide range of background odor environments.

2. Reviewers noted the need to better constrain the physiological impact of the recurrent axoaxonic synapses among CO_2-selective OSNs. In particular, they asked us to consider the diversity of acetylcholine’s actions, the possibility that these axoaxonic contacts are not purely excitatory, and whether ribbon-like presynaptic specializations might support volumetric rather than strictly point-to-point synaptic signaling. We address this first by providing new transcriptomic data showing ACh processing enzymes and AChR expression in Glomerulus 1 neurons (Supplementary Figure 9). Second, we provide new modeling results (in the reviewer response) showing that enhanced CO_2 detection enabled by recurrent connections is not entirely "free" - it comes with a potential increase in false alarm rates and reflects a circuit-level prioritization of detection over discrimination for this particular stimulus.

3. Reviewers raised concerns about the generality of our findings beyond a single connectome. Currently, virtually all connectomic studies, particularly of newly mapped species, present a single EM dataset. Remarkably, in fact, typically one EM dataset is the basis for multiple papers. For example, Phelps, et al. *Cell* (2021) [1] is the dataset used in multiple papers including [2–5];

Cardona, et al. *Plos Biol* (2010) [6] is the dataset used in [7–10]; Scheffer, et al. *Elife* (2020) [11] dataset is used in [12–14]; Takemura, et al. *bioRxiv* (2023) [15] is the dataset used in [16,17]; and Zheng, et al. *Cell* (2018) [18] is the dataset used in the *Nature* FlyWire Collection of papers (https://www.nature.com/collections/hgcfafejia) amongst others. We feel it is beyond the scope of this work to generate and analyze another connectomic dataset. These are both time consuming and costly to generate and rigorously analyze. One possibility that was raised is to perform a single-section EM study, but genetic labeling for direct assessment of cell types during EM in the mosquito remains an untested challenge, and we would need to complete another EM volume to identify the same glomerulus for analysis in a new sample. We validate our results by checking that our results exist on both sides of the brain, which is an internal control that readily detects random artifacts and provides greater confidence in our results.

Point-by-point response to reviewers
(reviewer comments in blue, our response in black text):

Reviewer #1 (Chih-Ying Su signed her report): In this study, Bao et al. characterize the early-stage connectome of the CO2 neural circuit in the mosquito antennal lobe. Using automated serial-section transmission EM, the authors reconstruct the circuitry of glomeruli innervated by the Aedes aegypti maxillary palp, including the CO2-responsive glomerulus and neighboring glomeruli that receive direct input from olfactory sensory neurons co-housed with the CO2-sensing neuron. Through a comparative analysis, the authors identify several circuit features specialized for CO2 processing, including (1) a high degree of recurrent synaptic connectivity among CO2 neurons accompanied by relatively sparse feedforward output, and (2) the presence of ribbon-like presynaptic structures at these recurrent synapses. Finally, the authors develop a computational circuit model to suggest that recurrent connectivity is required for robust CO2 detection.

Overall, this is an in-depth, high-quality connectomic study executed with clear technical excellence. The work reveals a distinctive circuit organization that likely underlies the salience of CO2 as a key host-seeking cue. The comparative framework, contrasting CO2- versus odor-processing glomeruli in mosquitoes, as well as comparing mosquito and Drosophila CO2 circuits, is particularly appealing and provides important insight at single-synapse resolution. The dataset itself is impressive and will be a valuable resource for the field.

While I have one major concern and several specific comments, addressing these points would further strengthen the clarity, interpretability, and impact of the study.

Major concern:
The modeling results in Figure 6 are informative only if divisive normalization operates in Glomerulus 1. Gain control via GABAB-mediated presynaptic inhibition is known to be highly heterogeneous across Drosophila glomeruli; notably, the V glomerulus expresses very low levels of GABAB receptors (Root et al., Neuron, 2008). This raises the possibility that divisive normalization may have minimal impact in the V glomerulus. To support the assumptions of the model, the authors need to directly assess GABAB receptor expression in Glomerulus 1 and other mosquito glomeruli via immunostaining. If Glomerulus 1 is not subject to divisive normalization (similar to the V glomerulus), the relevance of the modeling framework, and thus the conclusions drawn from Figure 6, would need to be reconsidered.

We thank the reviewer for raising this important point, which we address in two ways:

First, we have added a new analysis of single nucleus RNA sequencing data to address the concerns that $GABA_B$ receptors may not be present in the CO_2-sensing neurons. This transcriptomic data, included in Figure 6, shows expression levels of GABA receptors and GABA processing enzymes in sensory neurons, including in the CpA the neurons that detect CO_2. *Aedes aegypti* has 3 $GABA_B$ receptor orthologues, and both $GABA_BR1$ and $GABA_BR2$ are expressed in the CO_2-sensing neurons, as well as the GABA receptor RDL. None of the GABA processing enzymes are expressed, consistent with the model that the OSNs are excitatory cholinergic neurons that receive inhibitory input from elsewhere. In order to determine if the CO_2-sensitive neurons have similar levels of GABA receptor expression to other olfactory sensory neuron (OSN) types, we compared GABA receptor expression levels in the CO_2-sensing CpA neurons to GABA receptor expression levels in the CpB and CpC cells of the maxillary palps, as well as the average level in all maxillary palp OSNs and all antennal OSNs. We find comparable levels in the CpA cells to the other sensory neuron cell types. We also note that $GABA_BR1$ expression is higher than $GABA_BR2$ in *Aedes aegypti*, which may point to divergent functional roles of these receptors in this mosquito and *Drosophila melanogaster*, however that remains to be tested. This new data demonstrates that GABA receptors are expressed in the CO_2-sensing neurons, which makes divisive normalization through GABA plausible in Glomerulus 1 neurons. We were not able to perform immunostaining because there are few validated antibodies that cross react with *Aedes aegypti*, however, we hope that gene expression data will be sufficient to address this important point.

Second, we performed a sensitivity analysis to assess how our model's conclusions depend on the strength of divisive normalization. We introduced a parameter β (0 to 1) that scales the effective impact of lateral inhibition on Glomerulus 1, where β = 1 corresponds to the full normalization assumed in the original model and β = 0 corresponds to no normalization (as in the *Drosophila* V glomerulus). This allows us to evaluate whether recurrent connections are still beneficial if GABA_B-mediated inhibition is weak in Glomerulus 1.

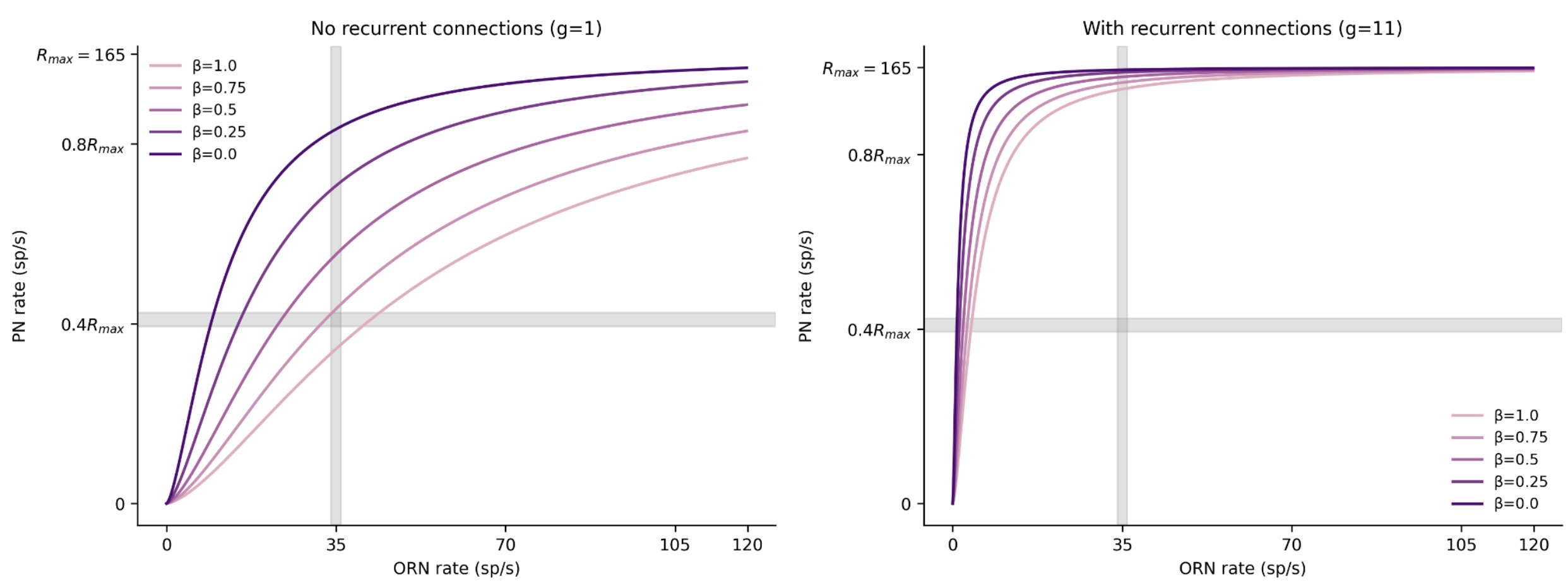

As shown above, reducing divisive normalization (lower β) shifts the PN response curves upward - weaker normalization means less suppression of Glomerulus 1 by background odors. In the left panel (g = 1, no recurrent connections), at low β values, PN responses at ORN = 35

sp/s exceed the detection threshold even without recurrent gain. In the right panel (g = 11, with recurrent connections), all curves comfortably exceed the detection threshold regardless of β.

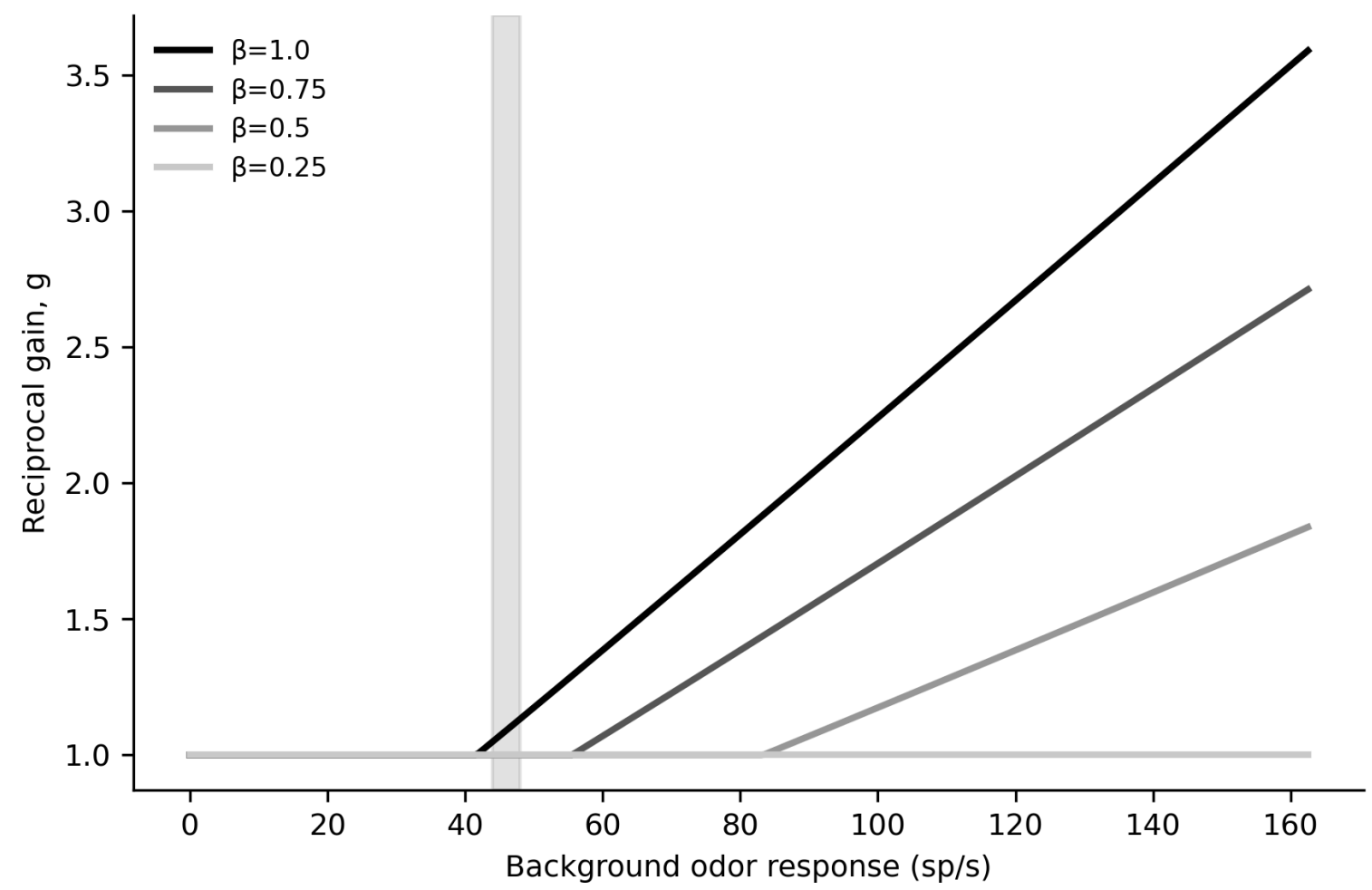

The figure above shows the minimum recurrent gain (g) required for detection across background odor strengths, for different levels of normalization. As divisive normalization weakens, the required g decreases - recurrent connections become less strictly necessary for detection. However, the measured g = 11 sits far above all curves at the empirical background level (gray band), indicating that the recurrent connections provide a large safety margin regardless of normalization strength. Furthermore, we note that the large measured value of g may itself be indirect evidence that divisive normalization is operating in Glomerulus 1. As discussed in our manuscript, one interpretation of the large g is that inhibition is significantly stronger than excitation in this circuit - which would imply that $GABA_B$-mediated lateral inhibition is indeed present and active, requiring substantial recurrent gain to overcome it. If normalization were weak or absent, a much smaller g would suffice for detection, which is inconsistent with the large number of recurrent connections we observe anatomically.

In summary, if Glomerulus 1 has weak $GABA_B$-mediated normalization, recurrent connections are not harmful — and if any normalization is present, they actively ensure robust CO_2 detection across a wide range of background odor environments.

Specific comments:

(1) Figure 1B and line 150: Replace "basiconic sensilla" with "capitate peg sensillum" (singular). Capitate peg sensilla and basiconic sensilla are distinct sensillum classes with different morphologies and pore architectures and should not be used interchangeably.

We have revised this in the figure and text.

(2) Line 138: Revise "innervated by the maxillary palp" to "innervated by olfactory sensory neurons of the maxillary palp."

We have revised this in the text.

(3) Fig 1: Consider reordering the figure panels so that Fig. 1I precedes Fig. 1H.

We considered reordering these panels, however, we felt it was clearer to present the cable length first, which is used to calculate cable density.

(4) Fig 1H: In addition to total cable length, quantifying and comparing branch number and branch order would help clarify whether the increased cable length reflects a greater number of branches or longer individual branches.

We have provided this additional analysis in two new figure panels in Supplementary Figure 5.

(5) Line 215: The statement "high concentration of CO2 is aversive" is misleading, as it implies that low concentrations are not aversive (but they are). In Drosophila, CO2 valence is determined primarily by the internal state of the animal rather than concentration per se (Breugel et al., Nature, 2018). This sentence should be revised accordingly.

Thank you for pointing this out. We have revised this in the text.

(6) The analyses presented in Figures 3 and 4 would be substantially strengthened by extension to mosquito Glomeruli 2 and 3. In addition, for the Drosophila comparison, it would be important to highlight glomeruli such as DM1, VA2, and DL1, which receive direct input from the neighbors of CO2 neurons (analogous to mosquito Glomeruli 2 and 3). This expanded comparison would allow a more rigorous assessment of which circuit features are specific to CO2 processing versus those shared with canonical odor-sensing glomeruli, and would sharpen the mosquito-fly comparison.

We provided this extended analysis in Supplementary Figures 7-8 for completeness, though do still prefer to focus the main figures on Glomerulus 1 for clarity and interpretiblity.

Reviewer #2: In this study, the authors characterize the connectivity architecture of the olfactory circuitry underlying CO_2 sensing in female Aedes aegypti mosquitoes. Because female mosquitoes rely on CO_2 detection to locate human hosts and serve as major vectors of disease, understanding the neurobiological basis of CO_2 sensing has important implications for mosquito-borne disease control. Using electron microscopy, the authors reconstruct the morphologies and synaptic connectivity of the three olfactory sensory neuron (OSN) types innervating the maxillary palp glomeruli, including the CO_2-sensitive channel (glomerulus 1) and two odorant-sensitive channels (glomeruli 2 and 3). They quantify synaptic connectivity within and between OSNs and downstream projection neurons (PNs), and perform comparative analyses using existing Drosophila melanogaster connectomic datasets. The authors report that CO_2-sensitive OSNs in mosquitoes exhibit high levels of OSN-OSN connectivity relative to odorant-sensitive OSNs and that their axo-axonal synapses onto each other are more numerous than their synapses onto olfactory projection neurons. Furthermore, CO_2-sensitive OSNs

contain ribbon-like synapses localized to lower-order axonal branches and associated with recurrent OSN-OSN contacts. This potentially unique cell biology is particularly striking. The authors' work on these points is convincing, the data is high quality and highly original, and this first foray into using EM connectomic reconstructions to interrogate circuit mechanisms in Aedes will have a strong influence on the field.

The authors go on to propose potential functions for these connections among CO2-sensing OSNs and for the ribbon synapse structure. Because of the nature of the data, which is static, the arguments in this part of the manuscript are weaker. We accept the limitations of the method and that augmenting the dataset with perturbation experiments or extensive functional imaging (as could be done in genetic model systems) is not necessarily feasible in this species. Indeed, the difficulty of such experiments in this species is a primary reason why the connectomic approach is likely to be so useful. We also appreciate the authors' care in being precise about what they can and cannot say with the existing data. In sum, the study is appropriate for publication in PLOS Biology after addressing our major points below, where we raise questions of interpretation and make suggestions of additional analyses that the authors should incorporate in revision.

Major Revisions:

1. The first point is conceptual: In their modeling, the authors treat axo-axonic, cholinergic synapses as simply excitatory. However, the physiological impact of acetylcholine will depend on what receptor is present in OSN axons. As shown by recent papers from Moshe Parnas's lab, axonal receipt of acetylcholine can be via different receptors than dendritic receipt and produce different physiological adjustments. If the authors can test which kind of receptors are in these axons, that would be great. If they can't, they should model the different possibilities and leave themselves open to the possibility that these connections may not be excitatory. It's possible there is other literature that we are unaware of that makes this unlikely, but either way it needs to be addressed head on.

We thank the reviewer for raising this important point. We have added a new analysis in Supplementary Figure 9 of single cell OSN transcriptomic data that shows the expression level of the acetylcholine receptors and processing enzymes. CO_2-responsive CpA OSNs express nicotinic, but not muscarinic, acetylcholine receptors. We compare them to the average population of OSNs in the antenna and maxillary palps, as well as to the CpB and CpC cells. *Aedes aegypti* nAChRalpha subunits 4, 5, 6, and 7 are expressed in CO_2-responsive OSNs (n.b. that the numbering does not indicate a 1-to-1 orthologue with *Drosophila melanogaster*. Rather, *Aedes aegypti* nAChRalpha4 is most closely related to *Drosophila melanogaster* nAChRalpha3/4, and nAChRalpha5, 6, and 7 are equally similar to *Drosophila melanogaster* nAChRalpha5/6/7). We note that the expression levels are low, but there are 4 subunits expressed at higher levels than in other OSN types. This expression of nAChRs but not mAChRs suggests recurrent connectivity mediated by ACh in Glomerulus 1 acts in an excitatory manner.

2. Second, in vertebrates, ribbon synapses can be involved in volume transmission of acetylcholine. It appears that in this species (and perhaps in insects in general?) synapses are predicted in EM based only on the presynaptic visual hallmark (the T-bar structure). Are there clear postsynaptic profiles? If so, are their structures the same or different between OSNs and OSNs (axo-axonal) versus OSNs and PNs

(axo-dendritic)? Is acetylcholine necessarily received in a localized way at these axons? If not, would this change the predicted circuit function?

Insect synapses are readily identifiable in EM using the following ultrastructural criteria: a presynaptic specialization (T-bar), presynaptic vesicles, a synaptic cleft, and a postsynaptic density (PSD) [19–21]. *Drosophila* synapses exhibit sufficient structural diversity to be predictive of neurotransmitter type, however, PSDs specifically are typically homogeneous (Supplementary Fig, 6 in ref. [21]) and aren't the synaptic feature differentiating synapse types[22]. Consistent with this, we find in that OSN-OSN axo-axonic and and OSN-PN axo-dendritic synapses also have these ultrastructural features in *Aedes aegypti*. Findings in *Drosophila* also support the hypothesis that such synapses are the structural substrate for local signalling of fast neurotransmitters like Ach. Together, our data supports the hypothesis that Ach acts in excitatory, point-to-point transmission between CO_2-selective OSNs.

Also please see response to (5) below.

3. The manuscript focuses on connectivity that is compartmentalized at specific neuronal structures, but doesn't show much anatomic data. The flat maps of branch order are useful, but the authors should also show the anatomically instantiated OSNs of interest and plot the locations of the synapses they make with one another, versus onto PN dendrites, and which of these are ribbon synapses.

We thank the reviewer for this suggestion. We have replotted the axons with different synapse types in Supplementary Fig. 3 to provide additional anatomical information and relationships between synapse types.

4. The authors do a nice job of using the other maxillary palp glomeruli as a comparison set within the Aedes data, which allows them to test that aspects of CO2 glomerulus connectivity are unique. The authors then go on to use a comparison to a Drosophila melanogaster connectomic dataset to ask if Aedes's CO2 glomerulus is unusual compared to olfactory glomeruli more generally. Because different EM connectomic datasets have different resolutions and have been reconstructed using different pipelines, our understanding is that they call synapses at different overall rates. We thus worry that ranking the Aedes glomeruli within the trajectory of glomeruli from a different dataset, from a different lab, isn't an apples-to-apples comparison. The authors know this from the inside-does the FAFB FlyWire reconstruction allow direct comparison with this Aedes volume? If they are confident the answer is yes, they should explain to the reader why this is the case. If no, then they need to explain how they're normalizing across the datasets. The recent preprint, "Invariant synaptic density across species" from Castro and Cardona could provide a starting point for normalization.

This is a good concern to raise and one that is becoming increasingly important as more comparative analysis is being done across datasets. Interestingly in Castro and Cardona, 2024 [23] the authors normalize and compare connectivity across species by computing synaptic density by normalizing synapse counts by cable length as well as tune synapse detection thresholds. We used a similar strategy (eg. Figs. 1M, 2F, 3C, 4C; Supplementary Figs. 5, 7-8).

In our case, we also have the additional benefit that both the FAFB-FlyWire and *Aedes* datasets were acquired with transmission electron microscopy and with similar serial-section thicknesses and signal-to-noise. This makes the datasets quite comparable in many respects. Our manual analysis was also comparable to analysis performed in the Janelia FAFB walled-garden [18] using CATMAID-based tracing and analysis, which were consistent and extended with FAFB-FlyWire [24].

To further examine this issue and make synapse counts comparable we estimated a cleft score for Flywire antennal lobe synapses with optimal and comparable accuracy, we manually inspected a sample of 19 feedforward OSN to PN synapses in fly glomereulus V. We found a cleft score of 65 that provided the highest f-score = 0.91, with precision = 0.88 and recall = 0.94. This means ~6% of manually annotated synapses are missed, with slightly more false positives. We therefore used a FAFB-Flywire cleft score of 65 across our analyses, which we explain in more detail in the Methods.

5. In the authors' model and conceptualization, is the enhanced detection of CO2 coming at the cost of doing some other olfactory thing worse? Or is it all upside?

This is an important question. In our model, recurrent connections in Glomerulus 1 amplify weak OSN signals to enhance CO_2 detection, but this does not come entirely without cost.

First, the large measured value of g may reflect a strong preference for detecting low levels of CO_2, even at the expense of false alarms. By amplifying weak inputs, recurrent connections lower the effective detection threshold, which increases sensitivity but also increases the likelihood that noise or transient fluctuations in ORN activity are misinterpreted as CO_2. This is a classic sensitivity-specificity trade-off. We clarify this in the Discussion:

> *By amplifying weak inputs, recurrent connections lower the effective detection threshold, which increases sensitivity, but also increases the risk of false-alarms from noise or transient fluctuations in OSN activity. This is a classic example of a sensitivity-specificity trade-off and may reflect a circuit-level prioritization of detection over discrimination for CO_2 in Ae. aegypti.*

Second, a recent analysis by Manoim-Wolkovitz et al. (2025) [25] demonstrated that when recurrent axo-axonic connections are present across all glomeruli, they can improve odor discrimination through pattern decorrelation, effectively enhancing the functional role of divisive inhibition. Our work demonstrates a complementary but distinct role: recurrent connections in a single, high-priority glomerulus for signal amplification rather than discrimination. These two computations - detection via amplification and discrimination via decorrelation - may represent competing demands. Dedicating recurrent gain to one glomerulus prioritizes detection of an ethologically critical cue (CO_2) but does not contribute to, and could in principle interfere with, broader odor discrimination. Further experimental investigation is necessary to understand how species-specific olfactory systems balance these two computations, perhaps as a function of ethological niche.

In summary, the enhanced CO_2 detection enabled by recurrent connections is not entirely "free" — it comes with a potential increase in false alarm rates and reflects a circuit-level prioritization of detection over discrimination for this particular stimulus.

6. In the same way the authors are circumspect in the main text about their conclusions, the title should be adjusted to more precisely reflect that "enabling sensitivity" is, at this point, a connectome-predicted hypothesis about circuit function.

We have revised the title to: “Connectivity supporting carbon dioxide sensitivity in the *Aedes aegypti* mosquito.” We are open to additional updates to the title if needed.

Minor Revisions

These suggestions may improve the presentation:

Figure 1B: The outline of the fly head and body prints very faintly. Increasing the line weight would improve visibility and overall figure clarity.

Thank you, we have updated the figure.

Figures 1K and 2D: It would aid reader orientation to include a direct label on the figure (e.g., "axogram"), rather than requiring readers to rely solely on the legend to interpret what is shown.

We have updated the figure.

Figure 1F: Some colors used to highlight EM structures in the OSN>OSN image are reused in the OSN>PN image but denote different structures. This reuse is potentially confusing. Using distinct, consistent color schemes across panels would improve clarity.

We have updated the figure.

Figure 1 Title: The phrase "chemosensory driven behaviors" should be removed from the figure title, as no behavioral data are presented in this figure.

We have updated the figure title.

Figures 3B-E: The color used for Drosophila glomerulus V does not sufficiently stand out from the colors representing other Drosophila glomeruli. Assigning a more salient color (as was done for mosquito glomerulus 1) would improve visual emphasis and interpretability.

We have updated the figure.

Introduction (Lines 62-63): The authors state that they sought to determine how CO_2 activation and sensitization mechanisms are reflected in neuronal circuitry and how this circuitry integrates CO_2 with other host cues. While the data address the first objective, the second objective is not explored in the current manuscript. For clarity and alignment with the presented data, the authors may consider revising or removing the latter motivation.

We feel the sentence provides a broader vision and rationale for our work, so we would prefer keeping it as it is, though if the reviewer feels strongly are open to updating it.

Line 105: The phrase "To examine the circuit mechanisms that drive CO_2 sensitivity…" may overstate the scope of the study. Revising this to "To examine the circuit architecture that characterizes CO_2 sensitivity…" would more accurately reflect the anatomical nature of the work and avoid implying functional testing.

We have updated the text to: "To examine the circuit architecture that underlies CO_2 sensitivity, we generated a synapse-resolution wiring diagram of the three glomeruli that are innervated by maxillary palp OSNs."

Reviewer #3: In this manuscript, Jialu Bao, Wei-Chung Lee and colleagues present a detailed neuroanatomical characterization of neural connectively within antennal lobe glomeruli innervated by maxillary palp-derived neurons in Aedes aegypti from a single female brain. Overall, the manuscript is clearly written, qualified in its wording and statements, and is a valuable contribution to the field. The data presented are suggestive that differences in morphology between different CO2 neurons in Ae. aegypti and D. melanogaster, may potentially underlie the unique behavioral effects of this stimulus in Ae. aegypti. Namely, the authors describe in Ae. aegypti recurrent OSN connectivity among CO2 neurons, which outnumber feedforward synapses; describe differences in the neuroanatomy of connectivity between CO2 neurons innervating Glomerulus 1 (Aedes aegypti) relative to the V-glomerulus (Drosophila melanogaster); and characterize putative ribbon-like synapses in Ae. aegypti CO2 neurons. This complements a recent study in PNAS that focuses on comparative dendritic and soma morphologies of CO2 neurons in Drosophila and Aedes aegypti (PMID: 41129220). Combined these studies suggest specialized morphology of the Ae. aegypti CO2 neuron.

Major Comments:

1. The partial connectome described here results from reconstructing a partial volume of the antennal lobe of a single female brain. Your findings are very interesting.

I understand that the work presented is probably a monumental effort in itself, but it still only represents a single replicate. The authors should provide additional targeted microscopy data presenting the generality of their findings to other replicate brains. For instance, can you document the existence of ribbon like synapses in a reduced subset of sections from Glomerulus 1 in other replicate brains?

I understand a full-blown replicated connectome is beyond the scope of this paper, but some targeted analyses should be possible to speak to the reproducibility of these findings. The need for replication (where it is not possible within a 1-3 month revision time frame) should be discussed.

We thank you for this important point. Please see Summary point 3 above. Currently, virtually all connectomic studies, particularly of newly mapped species use a single EM dataset. It is beyond the scope of this work to generate and analyze another connectomic dataset. We appreciate this caveat of connectomics in general, and are looking forward to a time when these types of datasets are cheaper and easier to collect.

2. With regards to this partial reconstruction of the innervation of Glomerulus 1, 2 and 3, you mention that you characterized 3 classes of neurons in your analysis - OSNs, uPNs and MGs. Are MGs categorized at local neurons? Given you did fully reconstruct the volumes of Glomerulus 1, 2 and 3, what type of MG connectivity did you observe between these glomeruli? This should be reported where possible and more clearly described in the methods.

To be conservative, with our partial dataset we do not differentiate between local neurons and multiglomerular projection neurons, but instead consider any neuron that extends beyond the home glomerulus a multiglomerular neuron. This was by design as we do not try to differentiate between these cell types and they are not the focus of this study and we anticipate future work will rigorously differentiate between such cell types in the antennal lobe as well as across the brain. We have added the following in the Methods:

> *Neurons not identified as OSNs or uPNs are referred to here as MGs. Operationally, MGs were reconstructed cells that extended beyond their home glomerulus. Most of these are likely LNs, because this cell type is the most numerous and broadly-arborizing of the MGs in the insect antennal lobe [26,27]. Additionally, some of these may be mPNs, or neurons that project into the antennal lobe from other regions.*

References

1. Phelps JS, Hildebrand DGC, Graham BJ, Kuan AT, Thomas LA, Nguyen TM, et al. Reconstruction of motor control circuits in adult Drosophila using automated transmission electron microscopy. Cell. 2021. doi:10.1016/j.cell.2020.12.013

2. Azevedo A, Lesser E, Phelps JS, Mark B, Elabbady L, Kuroda S, et al. Connectomic reconstruction of a female Drosophila ventral nerve cord. Nature. 2024. doi:10.1038/s41586-024-07389-x

3. Lesser E, Azevedo AW, Phelps JS, Elabbady L, Cook A, Syed DS, et al. Synaptic architecture of leg and wing premotor control networks in Drosophila. Nature. 2024. doi:10.1038/s41586-024-07600-z

4. Cheong HSJ, Boone KN, Bennett MM, Salman F, Ralston JD, Hatch K, et al. Organization of an ascending circuit that conveys flight motor state in Drosophila. Curr Biol. 2024;34: 1059–1075.e5.

5. Stürner T, Brooks P, Serratosa Capdevila L, Morris BJ, Javier A, Fang S, et al. Comparative connectomics of Drosophila descending and ascending neurons. Nature. 2025 [cited 4 May 2025]. doi:10.1038/s41586-025-08925-z

6. Cardona A, Saalfeld S, Preibisch S, Schmid B, Cheng A, Pulokas J, et al. An integrated micro- and macroarchitectural analysis of the Drosophila brain by computer-assisted serial section electron microscopy. PLoS Biol. 2010;8: e1000502.

7. Ohyama T, Schneider-Mizell CM, Fetter RD, Aleman JV, Franconville R, Rivera-Alba M, et al. A multilevel multimodal circuit enhances action selection in Drosophila. Nature. 2015;520: 633–639.

8. Jovanic T, Schneider-Mizell CM, Shao M, Masson J-B, Denisov G, Fetter RD, et al. Competitive Disinhibition Mediates Behavioral Choice and Sequences in Drosophila. Cell. 2016;167: 858–870.e19.

9. Eichler K, Li F, Litwin-Kumar A, Park Y, Andrade I, Schneider-Mizell CM, et al. The complete connectome of a learning and memory centre in an insect brain. Nature. 2017;548: 175–182.

10. Winding M, Pedigo BD, Barnes CL, Patsolic HG, Park Y, Kazimiers T, et al. The connectome of an insect brain. Science. 2023;379: eadd9330.

11. Scheffer LK, Xu CS, Januszewski M, Lu Z, Takemura S-Y, Hayworth KJ, et al. A connectome and analysis of the adult Drosophila central brain. Elife. 2020;9. doi:10.7554/eLife.57443

12. Li F, Lindsey JW, Marin EC, Otto N, Dreher M, Dempsey G, et al. The connectome of the adult Drosophila mushroom body provides insights into function. Elife. 2020;9. doi:10.7554/eLife.62576

13. Turner-Evans DB, Jensen KT, Ali S, Paterson T, Sheridan A, Ray RP, et al. The neuroanatomical ultrastructure and function of a biological ring attractor. Neuron. 2020;108: 145–163.e10.

14. Hulse BK, Haberkern H, Franconville R, Turner-Evans D, Takemura S-Y, Wolff T, et al. A connectome of the Drosophila central complex reveals network motifs suitable for flexible navigation and context-dependent action selection. Elife. 2021;10. doi:10.7554/eLife.66039

15. Takemura SY, Hayworth KJ, Huang GB. A Connectome of the Male Drosophila Ventral Nerve Cord. BioRxiv. 2023. Available: https://www.biorxiv.org/content/10.1101/2023.06.05.543757.abstract

16. Marin EC, Morris BJ, Stuerner T, Champion AS, Krzeminski D, Badalamente G, et al. Systematic annotation of a complete adult male Drosophila nerve cord connectome reveals principles of functional organisation. bioRxiv. 2023. doi:10.1101/2023.06.05.543407

17. Cheong HSJ, Eichler K, Stürner T, Asinof SK, Champion AS, Marin EC, et al. Transforming descending input into motor output: An analysis of the Drosophila Male Adult Nerve Cord connectome. 2025. doi:10.7554/elife.96084.2

18. Zheng Z, Lauritzen JS, Perlman E, Robinson CG, Nichols M, Milkie D, et al. A Complete Electron Microscopy Volume of the Brain of Adult Drosophila melanogaster. Cell. 2018;174: 730–743 e22.

19. Prokop A, Meinertzhagen IA. Development and structure of synaptic contacts in Drosophila. Semin Cell Dev Biol. 2006;17: 20–30.

20. Wagh DA, Rasse TM, Asan E, Hofbauer A, Schwenkert I, Dürrbeck H, et al. Bruchpilot, a protein with homology to ELKS/CAST, is required for structural integrity and function of synaptic active zones in Drosophila. Neuron. 2006;49: 833–844.

21. Tobin WF, Wilson RI, Lee W-CA. Wiring variations that enable and constrain neural computation in a sensory microcircuit. Elife. 2017;6. doi:10.7554/eLife.24838

22. Eckstein N, Bates AS, Champion A, Du M, Yin Y, Schlegel P, et al. Neurotransmitter classification from electron microscopy images at synaptic sites in Drosophila melanogaster. Cell. 2024;187: 2574–2594.e23.

23. Ferreira Castro A, Cardona A. Invariant synaptic density across species. bioRxiv. 2024. p. 2024.07.18.604056. doi:10.1101/2024.07.18.604056

24. Dorkenwald S, Matsliah A, Sterling AR, Schlegel P, Yu S-C, McKellar CE, et al. Neuronal wiring diagram of an adult brain. Nature. 2024;634: 124–138.

25. Manoim-Wolkovitz JE, Camchy T, Rozenfeld E, Chang H-H, Lerner H, Chou Y-H, et al. Nonlinear high-activity neuronal excitation enhances odor discrimination. Curr Biol. 2025;35: 1521–1538.e5.

26. Chou YH, Spletter ML, Yaksi E, Leong JC, Wilson RI, Luo L. Diversity and wiring variability of olfactory local interneurons in the Drosophila antennal lobe. Nat Neurosci. 2010;13: 439–449.

27. Lin S, Kao CF, Yu HH, Huang Y, Lee T. Lineage analysis of Drosophila lateral antennal lobe neurons reveals notch-dependent binary temporal fate decisions. PLoS Biol. 2012;10: e1001425.
