# Supplementary material for: Recurrent synapses between CO2-sensitive olfactory sensory neurons enable robust CO2 detection in Aedes aegypti mosquitoes
Source: PLoS Biol. 2026 Sep 10;24(9):e3003959. doi: 10.1371/journal.pbio.3003959 (PMC13561330; doi:10.1371/journal.pbio.3003959)
Supplement: Peer review history

# Editor report: Decision Letter 0

**DOI:** 10.1371/journal.pbio.3003959.r001

**Authors:** Christian Schnell, PhD

28 Jan 2026

Dear Dr Lee,

Thank you for submitting your manuscript entitled "Connectivity enabling carbon dioxide sensitivity in the Aedes aegypti mosquito" for consideration as a Research Article by PLOS Biology.

Your manuscript has now been evaluated by the PLOS Biology editorial staff as well as by an academic editor with relevant expertise and I am writing to let you know that we would like to send your submission out for external peer review.

However, before we can send your manuscript to reviewers, we need you to complete your submission by providing the metadata that is required for full assessment. To this end, please login to Editorial Manager where you will find the paper in the 'Submissions Needing Revisions' folder on your homepage. Please click 'Revise Submission' from the Action Links and complete all additional questions in the submission questionnaire.

Once your full submission is complete, your paper will undergo a series of checks in preparation for peer review. After your manuscript has passed the checks it will be sent out for review. To provide the metadata for your submission, please Login to Editorial Manager (https://www.editorialmanager.com/pbiology) within two working days, i.e. by Jan 30 2026 11:59PM.

If your manuscript has been previously peer-reviewed at another journal, PLOS Biology is willing to work with those reviews in order to avoid re-starting the process. Submission of the previous reviews is entirely optional and our ability to use them effectively will depend on the willingness of the previous journal to confirm the content of the reports and share the reviewer identities. Please note that we reserve the right to invite additional reviewers if we consider that additional/independent reviewers are needed, although we aim to avoid this as far as possible. In our experience, working with previous reviews does save time.

If you would like us to consider previous reviewer reports, please edit your cover letter to let us know and include the name of the journal where the work was previously considered and the manuscript ID it was given. In addition, please upload a response to the reviews as a 'Prior Peer Review' file type, which should include the reports in full and a point-by-point reply detailing how you have or plan to address the reviewers' concerns.

During the process of completing your manuscript submission, you will be invited to opt-in to posting your pre-review manuscript as a bioRxiv preprint. Visit http://journals.plos.org/plosbiology/s/preprints for full details. If you consent to posting your current manuscript as a preprint, please upload a single Preprint PDF.

Feel free to email us at plosbiology@plos.org if you have any queries relating to your submission.

Kind regards,

Christian

Christian Schnell, PhD

Senior Editor

PLOS Biology

cschnell@plos.org

# Peer review: Decision Letter 1

**DOI:** 10.1371/journal.pbio.3003959.r002

**Authors:** Christian Schnell, PhD

11 Mar 2026

Dear Wei,

Thank you for your patience while your manuscript "Connectivity enabling carbon dioxide sensitivity in the Aedes aegypti mosquito" was peer-reviewed at PLOS Biology. It has now been evaluated by the PLOS Biology editors, an Academic Editor with relevant expertise, and by several independent reviewers.

In light of the reviews, which you will find at the end of this email, we would like to invite you to revise the work to thoroughly address the reviewers' reports.

As you will see below, the reviewers agree that the study is overall well executed and provides very important insights. Among the concerns raised by the reviewers, the replicability, the identity of the involved neurotransmitter receptors and the modelling are the most important concerns which will need to be addressed in a revision.

I would strongly encourage to get in touch with me via email when you have developed a revision plan, so I can discuss your revision plan with the academic editor and provide further guidance regarding the proposed revisions.

Given the extent of revision needed, we cannot make a decision about publication until we have seen the revised manuscript and your response to the reviewers' comments. Your revised manuscript is likely to be sent for further evaluation by all or a subset of the reviewers.

In addition to these revisions, you will need to complete some formatting changes, which you will receive in a follow up email. A member of our team will be in touch with a set of requests shortly.

We expect to receive your revised manuscript within 3 months. Please email us (plosbiology@plos.org) if you have any questions or concerns, or would like to request an extension.

At this stage, your manuscript remains formally under active consideration at our journal; please notify us by email if you do not intend to submit a revision so that we may withdraw it.

**IMPORTANT - SUBMITTING YOUR REVISION**

Your revisions should address the specific points made by each reviewer. Please submit the following files along with your revised manuscript:

1. A 'Response to Reviewers' file - this should detail your responses to the editorial requests, present a point-by-point response to all of the reviewers' comments, and indicate the changes made to the manuscript.

*NOTE: In your point-by-point response to the reviewers, please provide the full context of each review. Do not selectively quote paragraphs or sentences to reply to. The entire set of reviewer comments should be present in full and each specific point should be responded to individually, point by point.

You should also cite any additional relevant literature that has been published since the original submission and mention any additional citations in your response.

2. In addition to a clean copy of the manuscript, please also upload a 'track-changes' version of your manuscript that specifies the edits made. This should be uploaded as a "Revised Article with Changes Highlighted" file type.

*Re-submission Checklist*

When you are ready to resubmit your revised manuscript, please refer to this re-submission checklist: https://plos.io/Biology_Checklist

To submit a revised version of your manuscript, please go to https://www.editorialmanager.com/pbiology/ and log in as an Author. Click the link labelled 'Submissions Needing Revision' where you will find your submission record.

Please make sure to read the following important policies and guidelines while preparing your revision:

*Published Peer Review*

Please note while forming your response, if your article is accepted, you may have the opportunity to make the peer review history publicly available. The record will include editor decision letters (with reviews) and your responses to reviewer comments. If eligible, we will contact you to opt in or out. Please see here for more details:

https://blogs.plos.org/plos/2019/05/plos-journals-now-open-for-published-peer-review/

*PLOS Data Policy*

Please note that as a condition of publication PLOS' data policy (http://journals.plos.org/plosbiology/s/data-availability) requires that you make available all data used to draw the conclusions arrived at in your manuscript. If you have not already done so, you must include any data used in your manuscript either in appropriate repositories, within the body of the manuscript, or as supporting information (N.B. this includes any numerical values that were used to generate graphs, histograms etc.). For an example see here: http://www.plosbiology.org/article/info%3Adoi%2F10.1371%2Fjournal.pbio.1001908#s5

*Blot and Gel Data Policy*

We require the original, uncropped and minimally adjusted images supporting all blot and gel results reported in an article's figures or Supporting Information files. We will require these files before a manuscript can be accepted so please prepare them now, if you have not already uploaded them. Please carefully read our guidelines for how to prepare and upload this data: https://journals.plos.org/plosbiology/s/figures#loc-blot-and-gel-reporting-requirements

*Protocols deposition*

To enhance the reproducibility of your results, we recommend that if applicable you deposit your laboratory protocols in protocols.io, where a protocol can be assigned its own identifier (DOI) such that it can be cited independently in the future. Additionally, PLOS ONE offers an option for publishing peer-reviewed Lab Protocol articles, which describe protocols hosted on protocols.io. Read more information on sharing protocols at https://plos.org/protocols?utm_medium=editorial-email&utm_source=authorletters&utm_campaign=protocols

Thank you again for your submission to our journal. We hope that our editorial process has been constructive thus far, and we welcome your feedback at any time. Please don't hesitate to contact us if you have any questions or comments.

Sincerely,

Christian

Christian Schnell, PhD

Senior Editor

PLOS Biology

cschnell@plos.org

------------------------------------

REVIEWS:

Reviewer #1 (Chih-Ying Su signed her report): In this study, Bao et al. characterize the early-stage connectome of the CO2 neural circuit in the mosquito antennal lobe. Using automated serial-section transmission EM, the authors reconstruct the circuitry of glomeruli innervated by the Aedes aegypti maxillary palp, including the CO2-responsive glomerulus and neighboring glomeruli that receive direct input from olfactory sensory neurons co-housed with the CO2-sensing neuron. Through a comparative analysis, the authors identify several circuit features specialized for CO2 processing, including (1) a high degree of recurrent synaptic connectivity among CO2 neurons accompanied by relatively sparse feedforward output, and (2) the presence of ribbon-like presynaptic structures at these recurrent synapses. Finally, the authors develop a computational circuit model to suggest that recurrent connectivity is required for robust CO2 detection.

Overall, this is an in-depth, high-quality connectomic study executed with clear technical excellence. The work reveals a distinctive circuit organization that likely underlies the salience of CO2 as a key host-seeking cue. The comparative framework, contrasting CO2- versus odor-processing glomeruli in mosquitoes, as well as comparing mosquito and Drosophila CO2 circuits, is particularly appealing and provides important insight at single-synapse resolution. The dataset itself is impressive and will be a valuable resource for the field.

While I have one major concern and several specific comments, addressing these points would further strengthen the clarity, interpretability, and impact of the study.

Major concern:

The modeling results in Figure 6 are informative only if divisive normalization operates in Glomerulus 1. Gain control via GABAB-mediated presynaptic inhibition is known to be highly heterogeneous across Drosophila glomeruli; notably, the V glomerulus expresses very low levels of GABAB receptors (Root et al., Neuron, 2008). This raises the possibility that divisive normalization may have minimal impact in the V glomerulus. To support the assumptions of the model, the authors need to directly assess GABAB receptor expression in Glomerulus 1 and other mosquito glomeruli via immunostaining. If Glomerulus 1 is not subject to divisive normalization (similar to the V glomerulus), the relevance of the modeling framework, and thus the conclusions drawn from Figure 6, would need to be reconsidered.

Specific comments:

(1)Figure 1B and line 150: Replace "basiconic sensilla" with "capitate peg sensillum" (singular). Capitate peg sensilla and basiconic sensilla are distinct sensillum classes with different morphologies and pore architectures and should not be used interchangeably.

(2)Line 138: Revise "innervated by the maxillary palp" to "innervated by olfactory sensory neurons of the maxillary palp."

(3)Fig 1: Consider reordering the figure panels so that Fig. 1I precedes Fig. 1H.

(4)Fig 1H: In addition to total cable length, quantifying and comparing branch number and branch order would help clarify whether the increased cable length reflects a greater number of branches or longer individual branches.

(5)Line 215: The statement "high concentration of CO2 is aversive" is misleading, as it implies that low concentrations are not aversive (but they are). In Drosophila, CO2 valence is determined primarily by the internal state of the animal rather than concentration per se (Breugel et al., Nature, 2018). This sentence should be revised accordingly.

(6)The analyses presented in Figures 3 and 4 would be substantially strengthened by extension to mosquito Glomeruli 2 and 3. In addition, for the Drosophila comparison, it would be important to highlight glomeruli such as DM1, VA2, and DL1, which receive direct input from the neighbors of CO2 neurons (analogous to mosquito Glomeruli 2 and 3). This expanded comparison would allow a more rigorous assessment of which circuit features are specific to CO2 processing versus those shared with canonical odor-sensing glomeruli, and would sharpen the mosquito-fly comparison.

Reviewer #2: In this study, the authors characterize the connectivity architecture of the olfactory circuitry underlying CO₂ sensing in female Aedes aegypti mosquitoes. Because female mosquitoes rely on CO₂ detection to locate human hosts and serve as major vectors of disease, understanding the neurobiological basis of CO₂ sensing has important implications for mosquito-borne disease control. Using electron microscopy, the authors reconstruct the morphologies and synaptic connectivity of the three olfactory sensory neuron (OSN) types innervating the maxillary palp glomeruli, including the CO₂-sensitive channel (glomerulus 1) and two odorant-sensitive channels (glomeruli 2 and 3). They quantify synaptic connectivity within and between OSNs and downstream projection neurons (PNs), and perform comparative analyses using existing Drosophila melanogaster connectomic datasets. The authors report that CO₂-sensitive OSNs in mosquitoes exhibit high levels of OSN-OSN connectivity relative to odorant-sensitive OSNs and that their axo-axonal synapses onto each other are more numerous than their synapses onto olfactory projection neurons. Furthermore, CO₂-sensitive OSNs contain ribbon-like synapses localized to lower-order axonal branches and associated with recurrent OSN-OSN contacts. This potentially unique cell biology is particularly striking. The authors' work on these points is convincing, the data is high quality and highly original, and this first foray into using EM connectomic reconstructions to interrogate circuit mechanisms in Aedes will have a strong influence on the field.

The authors go on to propose potential functions for these connections among CO2-sensing OSNs and for the ribbon synapse structure. Because of the nature of the data, which is static, the arguments in this part of the manuscript are weaker. We accept the limitations of the method and that augmenting the dataset with perturbation experiments or extensive functional imaging (as could be done in genetic model systems) is not necessarily feasible in this species. Indeed, the difficulty of such experiments in this species is a primary reason why the connectomic approach is likely to be so useful. We also appreciate the authors' care in being precise about what they can and cannot say with the existing data. In sum, the study is appropriate for publication in PLOS Biology after addressing our major points below, where we raise questions of interpretation and make suggestions of additional analyses that the authors should incorporate in revision.

Major Revisions:

1. The first point is conceptual: In their modeling, the authors treat axo-axonic, cholinergic synapses as simply excitatory. However, the physiological impact of acetylcholine will depend on what receptor is present in OSN axons. As shown by recent papers from Moshe Parnas's lab, axonal receipt of acetylcholine can be via different receptors than dendritic receipt and produce different physiological adjustments. If the authors can test which kind of receptors are in these axons, that would be great. If they can't, they should model the different possibilities and leave themselves open to the possibility that these connections may not be excitatory. It's possible there is other literature that we are unaware of that makes this unlikely, but either way it needs to be addressed head on.

2. Second, in vertebrates, ribbon synapses can be involved in volume transmission of acetylcholine. It appears that in this species (and perhaps in insects in general?) synapses are predicted in EM based only on the presynaptic visual hallmark (the T-bar structure). Are there clear postsynaptic profiles? If so, are their structures the same or different between OSNs and OSNs (axo-axonal) versus OSNs and PNs (axo-dendritic)? Is acetylcholine necessarily received in a localized way at these axons? If not, would this change the predicted circuit function?

3. The manuscript focuses on connectivity that is compartmentalized at specific neuronal structures, but doesn't show much anatomic data. The flat maps of branch order are useful, but the authors should also show the anatomically instantiated OSNs of interest and plot the locations of the synapses they make with one another, versus onto PN dendrites, and which of these are ribbon synapses.

4. The authors do a nice job of using the other maxillary palp glomeruli as a comparison set within the Aedes data, which allows them to test that aspects of CO2 glomerulus connectivity are unique. The authors then go on to use a comparison to a Drosophila melanogaster connectomic dataset to ask if Aedes's CO2 glomerulus is unusual compared to olfactory glomeruli more generally. Because different EM connectomic datasets have different resolutions and have been reconstructed using different pipelines, our understanding is that they call synapses at different overall rates. We thus worry that ranking the Aedes glomeruli within the trajectory of glomeruli from a different dataset, from a different lab, isn't an apples-to-apples comparison. The authors know this from the inside-does the FAFB FlyWire reconstruction allow direct comparison with this Aedes volume? If they are confident the answer is yes, they should explain to the reader why this is the case. If no, then they need to explain how they're normalizing across the datasets. The recent preprint, "Invariant synaptic density across species" from Castro and Cardona could provide a starting point for normalization.

5. In the authors' model and conceptualization, is the enhanced detection of CO2 coming at the cost of doing some other olfactory thing worse? Or is it all upside?

6. In the same way the authors are circumspect in the main text about their conclusions, the title should be adjusted to more precisely reflect that "enabling sensitivity" is, at this point, a connectome-predicted hypothesis about circuit function.

Minor Revisions

These suggestions may improve the presentation:

Figure 1B: The outline of the fly head and body prints very faintly. Increasing the line weight would improve visibility and overall figure clarity.

Figures 1K and 2D: It would aid reader orientation to include a direct label on the figure (e.g., "axogram"), rather than requiring readers to rely solely on the legend to interpret what is shown.

Figure 1F: Some colors used to highlight EM structures in the OSN>OSN image are reused in the OSN>PN image but denote different structures. This reuse is potentially confusing. Using distinct, consistent color schemes across panels would improve clarity.

Figure 1 Title: The phrase "chemosensory driven behaviors" should be removed from the figure title, as no behavioral data are presented in this figure.

Figures 3B-E: The color used for Drosophila glomerulus V does not sufficiently stand out from the colors representing other Drosophila glomeruli. Assigning a more salient color (as was done for mosquito glomerulus 1) would improve visual emphasis and interpretability.

Introduction (Lines 62-63): The authors state that they sought to determine how CO₂ activation and sensitization mechanisms are reflected in neuronal circuitry and how this circuitry integrates CO₂ with other host cues. While the data address the first objective, the second objective is not explored in the current manuscript. For clarity and alignment with the presented data, the authors may consider revising or removing the latter motivation.

Line 105: The phrase "To examine the circuit mechanisms that drive CO₂ sensitivity…" may overstate the scope of the study. Revising this to "To examine the circuit architecture that characterizes CO₂ sensitivity…" would more accurately reflect the anatomical nature of the work and avoid implying functional testing.

Reviewer #3: In this manuscript, Jialu Bao, Wei-Chung Lee and colleagues present a detailed neuroanatomical characterization of neural connectively within antennal lobe glomeruli innervated by maxillary palp-derived neurons in Aedes aegypti from a single female brain. Overall, the manuscript is clearly written, qualified in its wording and statements, and is a valuable contribution to the field. The data presented are suggestive that differences in morphology between different CO2 neurons in Ae. aegypti and D. melanogaster, may potentially underlie the unique behavioral effects of this stimulus in Ae. aegypti. Namely, the authors describe in Ae. aegypti recurrent OSN connectivity among CO2 neurons, which outnumber feedforward synapses; describe differences in the neuroanatomy of connectivity between CO2 neurons innervating Glomerulus 1 (Aedes aegypti) relative to the V-glomerulus (Drosophila melanogaster); and characterize putative ribbon-like synapses in Ae. aegypti CO2 neurons. This complements a recent study in PNAS that focuses on comparative dendritic and soma morphologies of CO2 neurons in Drosophila and Aedes aegypti (PMID: 41129220). Combined these studies suggest specialized morphology of the Ae. aegypti CO2 neuron.

Major Comments:

1. The partial connectome described here results from reconstructing a partial volume of the antennal lobe of a single female brain. Your findings are very interesting.

I understand that the work presented is probably a monumental effort in itself, but it still only represents a single replicate. The authors should provide additional targeted microscopy data presenting the generality of their findings to other replicate brains. For instance, can you document the existence of ribbon like synapses in a reduced subset of sections from Glomerulus 1 in other replicate brains?

I understand a full-blown replicated connectome is beyond the scope of this paper, but some targeted analyses should be possible to speak to the reproducibility of these findings. The need for replication (where it is not possible within a 1-3 month revision time frame) should be discussed.

2. With regards to this partial reconstruction of the innervation of Glomerulus 1, 2 and 3, you mention that you characterized 3 classes of neurons in your analysis - OSNs, uPNs and MGs. Are MGs categorized at local neurons? Given you did fully reconstruct the volumes of Glomerulus 1, 2 and 3, what type of MG connectivity did you observe between these glomeruli? This should be reported where possible and more clearly described in the methods.

# Author comment: Author response to Decision Letter 2

**DOI:** 10.1371/journal.pbio.3003959.r003

11 Jun 2026

Attachment Submitted filename: aedesAL_reviewerResponse.pdf

# Peer review: Decision Letter 2

**DOI:** 10.1371/journal.pbio.3003959.r004

**Authors:** Christian Schnell, PhD

1 Jul 2026

Dear Wei,

Thank you for your patience while we considered your revised manuscript "Connectivity supporting carbon dioxide sensitivity in the Aedes aegypti mosquito" for publication as a Research Article at PLOS Biology. This revised version of your manuscript has been evaluated by the PLOS Biology editors, the Academic Editor and two of the original reviewers.

Based on the reviews and on our Academic Editor's assessment of your revision, we are likely to accept this manuscript for publication, provided you satisfactorily address the following data and other policy-related requests:

* We would like to suggest a different title to improve its accessibility for our broad audience: "Recurrent synapses between CO2-sensitive olfactory sensory neurons are necessary for robust CO2 detection in Aedes aegypti mosquitoes"

* DATA POLICY:

You may be aware of the PLOS Data Policy, which requires that all data be made available without restriction: http://journals.plos.org/plosbiology/s/data-availability. For more information, please also see this editorial: http://dx.doi.org/10.1371/journal.pbio.1001797

Note that we do not require all raw data. Rather, we ask that all individual quantitative observations that underlie the data summarized in the figures and results of your paper be made available in one of the following forms:

1) Supplementary files (e.g., excel). Please ensure that all data files are uploaded as 'Supporting Information' and are invariably referred to (in the manuscript, figure legends, and the Description field when uploading your files) using the following format verbatim: S1 Data, S2 Data, etc. Multiple panels of a single or even several figures can be included as multiple sheets in one excel file that is saved using exactly the following convention: S1_Data.xlsx (using an underscore).

2) Deposition in a publicly available repository. Please also provide the accession code or a reviewer link so that we may view your data before publication.

Regardless of the method selected, please ensure that you provide the individual numerical values that underlie the summary data displayed in the following figure panels as they are essential for readers to assess your analysis and to reproduce it: 1HLMN, 2EFGH, 3BCDE, 4BCD, S5ABDEFG, S7ABC, S8ABC and S9BC.

NOTE: the numerical data provided should include all replicates AND the way in which the plotted mean and errors were derived (it should not present only the mean/average values).

Please also ensure that figure legends in your manuscript include information on where the underlying data can be found, and ensure your supplemental data file/s has a legend.

Please ensure that your Data Statement in the submission system accurately describes where your data can be found.

* CODE POLICY

Per journal policy, if you have generated any custom code during the course of this investigation, please make it available without restrictions. Please ensure that the code is sufficiently well documented and reusable, and that your Data Statement in the Editorial Manager submission system accurately describes where your code can be found. More information on our Code Policy, what and how to share can be found here: https://journals.plos.org/plosbiology/s/code-availability

Please note that we cannot accept sole deposition of code in GitHub, as this could be changed after publication. However, you can archive this version of your publicly available GitHub code to Zenodo. Once you do this, it will generate a DOI number, which you will need to provide in the Data Accessibility Statement (you are welcome to also provide the GitHub access information). See the process for doing this here: https://docs.github.com/en/repositories/archiving-a-github-repository/referencing-and-citing-content

As you address these items, please take this last chance to review your reference list to ensure that it is complete and correct. If you have cited papers that have been retracted, please include the rationale for doing so in the manuscript text, or remove these references and replace them with relevant current references. Any changes to the reference list should be mentioned in the cover letter that accompanies your revised manuscript.

In addition to these revisions, you may need to complete some formatting changes, which you will receive in a follow up email. A member of our team will be in touch with a set of requests shortly. If you do not receive a separate email within a few days, please assume that checks have been completed, and no additional changes are required.

We expect to receive your revised manuscript within two weeks.

To submit your revision, please go to https://www.editorialmanager.com/pbiology/ and log in as an Author. Click the link labelled 'Submissions Needing Revision' to find your submission record. Your revised submission must include the following:

- a cover letter that should detail your responses to any editorial requests, if applicable, and whether changes have been made to the reference list

- a Response to Reviewers file that provides a detailed response to the reviewers' comments (if applicable, if not applicable please do not delete your existing 'Response to Reviewers' file.)

- a track-changes file indicating any changes that you have made to the manuscript.

NOTE: If Supporting Information files are included with your article, note that these are not copyedited and will be published as they are submitted. Please ensure that these files are legible and of high quality (at least 300 dpi) in an easily accessible file format. For this reason, please be aware that any references listed in an SI file will not be indexed. For more information, see our Supporting Information guidelines:

https://journals.plos.org/plosbiology/s/supporting-information

*Published Peer Review History*

Please note that you may have the opportunity to make the peer review history publicly available. The record will include editor decision letters (with reviews) and your responses to reviewer comments. If eligible, we will contact you to opt in or out. Please see here for more details:

https://plos.org/published-peer-review-history/

*Press*

Should you, your institution's press office or the journal office choose to press release your paper, please ensure you have opted out of Early Article Posting on the submission form. We ask that you notify us as soon as possible if you or your institution is planning to press release the article.

*Protocols deposition*

To enhance the reproducibility of your results, we recommend that if applicable you deposit your laboratory protocols in protocols.io, where a protocol can be assigned its own identifier (DOI) such that it can be cited independently in the future. Additionally, PLOS ONE offers an option for publishing peer-reviewed Lab Protocol articles, which describe protocols hosted on protocols.io. Read more information on sharing protocols at https://plos.org/protocols?utm_medium=editorial-email&utm_source=authorletters&utm_campaign=protocols

Please do not hesitate to contact me should you have any questions.

Sincerely,

Christian

Christian Schnell, PhD

Senior Editor

cschnell@plos.org

PLOS Biology

------------------------------------------------------------------------

Reviewer remarks:

Reviewer #1 (Chih-Ying Su signed her report): The revisions have adequately addressed my previous concerns.

Reviewer #2: The authors have addressed all my previous points and I enthusiastically support publication.

# Author comment: Author response to Decision Letter 3

**DOI:** 10.1371/journal.pbio.3003959.r005

21 Jul 2026

Attachment Submitted filename: aedesAL_reviewerResponse_auresp_3.pdf

# Editor report: Decision Letter 3

**DOI:** 10.1371/journal.pbio.3003959.r006

**Authors:** Christian Schnell, PhD

5 Aug 2026

Dear Wei-Chung,

Thank you for the submission of your revised Research Article "Recurrent synapses between CO2-sensitive olfactory sensory neurons necessary for robust CO2 detection in Aedes aegypti mosquitoes" for publication in PLOS Biology. On behalf of my colleagues and the Academic Editor, Bing Ye, I am pleased to say that we can in principle accept your manuscript for publication, provided you address any remaining formatting and reporting issues. These will be detailed in an email you should receive within 2-3 business days from our colleagues in the journal operations team; no action is required from you until then. Please note that we will not be able to formally accept your manuscript and schedule it for publication until you have completed any requested changes.

Please take a minute to log into Editorial Manager at http://www.editorialmanager.com/pbiology/, click the "Update My Information" link at the top of the page, and update your user information to ensure an efficient production process.

PRESS

We frequently collaborate with press offices. If your institution or institutions have a press office, please notify them about your upcoming paper at this point, to enable them to help maximize its impact. If the press office is planning to promote your findings, we would be grateful if they could coordinate with biologypress@plos.org. If you have previously opted in to the early version process, we ask that you notify us immediately of any press plans so that we may opt out on your behalf.

We also ask that you take this opportunity to read our Embargo Policy regarding the discussion, promotion and media coverage of work that is yet to be published by PLOS. As your manuscript is not yet published, it is bound by the conditions of our Embargo Policy. Please be aware that this policy is in place both to ensure that any press coverage of your article is fully substantiated and to provide a direct link between such coverage and the published work. For full details of our Embargo Policy, please visit http://www.plos.org/about/media-inquiries/embargo-policy/.

Thank you again for choosing PLOS Biology for publication and supporting Open Access publishing. We look forward to publishing your study.

Sincerely,

Christian

Christian Schnell, PhD

Senior Editor

PLOS Biology

cschnell@plos.org
